# Risk of Ovarian Cancer and Inherited Variants in Relapse-Associated Genes

**DOI:** 10.1371/journal.pone.0008884

**Published:** 2010-01-27

**Authors:** Abraham Peedicayil, Robert A. Vierkant, Lynn C. Hartmann, Brooke L. Fridley, Zachary S. Fredericksen, Kristin L. White, Elaine A. Elliott, Catherine M. Phelan, Ya-Yu Tsai, Andrew Berchuck, Edwin S. Iversen, Fergus J. Couch, Prema Peethamabaran, Melissa C. Larson, Kimberly R. Kalli, Matthew L. Kosel, Vijayalakshmi Shridhar, David N. Rider, Mark Liebow, Julie M. Cunningham, Joellen M. Schildkraut, Thomas A. Sellers, Ellen L. Goode

**Affiliations:** 1 Mayo Clinic College of Medicine, Rochester, Minnesota, United States of America; 2 H. Lee Moffitt Cancer Research Institute, Tampa, Florida, United States of America; 3 Duke University, Durham, North Carolina, United States of America; University of Barcelona, Spain

## Abstract

**Background:**

We previously identified a panel of genes associated with outcome of ovarian cancer. The purpose of the current study was to assess whether variants in these genes correlated with ovarian cancer risk.

**Methods and Findings:**

Women with and without invasive ovarian cancer (749 cases, 1,041 controls) were genotyped at 136 single nucleotide polymorphisms (SNPs) within 13 candidate genes. Risk was estimated for each SNP and for overall variation within each gene. At the gene-level, variation within *MSL1* (male-specific lethal-1 homolog) was associated with risk of serous cancer (p = 0.03); haplotypes within *PRPF31* (PRP31 pre-mRNA processing factor 31 homolog) were associated with risk of invasive disease (p = 0.03). *MSL1* rs7211770 was associated with decreased risk of serous disease (OR 0.81, 95% CI 0.66–0.98; p = 0.03). SNPs in *MFSD7*, *BTN3A3*, *ZNF200*, *PTPRS*, and *CCND1A* were inversely associated with risk (p<0.05), and there was increased risk at *HEXIM1* rs1053578 (p = 0.04, OR 1.40, 95% CI 1.02–1.91).

**Conclusions:**

Tumor studies can reveal novel genes worthy of follow-up for cancer susceptibility. Here, we found that inherited markers in the gene encoding MSL1, part of a complex that modifies the histone H4, may decrease risk of invasive serous ovarian cancer.

## Introduction

Worldwide, there are approximately 125,000 deaths each year due to ovarian cancer [1]; increased understanding of factors related to its outcome and etiology should reduce the burden of this disease. We previously reported results of tumor mRNA expression studies which suggested that altered expression of a particular set of genes predicted response to chemotherapy among women with advanced-stage high-grade epithelial ovarian cancer [2]. These genes included *SF3A3*, *MFSD7* (formerly known as *FLJ22269*), *ID4*, *BTN3A3*, *OSGIN2* (formerly known as *C8orf1*), *FARP1*, *PRKCH*, *C15orf15*, *ZNF200*, *MSL1* (formerly known as *LOC339287*), *HEXIM1* (formerly known as *HIS1*), *PTPRS*, *CC2D1A* (formerly known as *FLJ20241*), and *PRPF31*. Expression levels differed among tumors from women with differing outcomes; namely, in combination, expression of these genes predicted early relapse (<21 months) after optimal surgery and platinum-paclitaxel chemotherapy with an accuracy of 86% and positive predictive value of 95% [3], [2].

The etiology of ovarian cancer is known to be complex and, at least in part, includes inherited susceptibility factors. Mutations in *BRCA1*, *BRCA2*, *MLH1*, and *MSH2* account for approximately 50% of familial ovarian cancer [4], [5], and remaining cases with a family history are likely due to combinations of multiple alleles conferring low to moderate penetrant susceptibility [6], [7] such as variants in *BNC2* [8] and, possibly, *TP53* [9], *CDKN2A* [10], *CDKN1B* [10], and *AURKA* [11]. As a complement to genome-wide searches, a useful approach for the identification of additional low-risk alleles is the study of highly-informative inherited variants in candidate genes identified from tumor expression studies. To assess whether variation in genes with differing expression levels by outcome influenced risk of ovarian cancer, we conducted a case-control analysis of inherited variants in genes in the predictive model mentioned above [2] as well as in *HTRA1* (encoding the serine protease HtrA1) which we have shown is down-regulated in a majority of ovarian tumors [12], [13] and has a key role in apoptosis [13]. As the first examination of germline variation in this novel set of genes (Table 1), we aimed to more broadly elucidate their role in epithelial ovarian carcinogenic processes.

**Table 1:** Candidate genes of interest.

Chr.	Gene Name	Description	GeneID	Refseq	Start (bp)	Size (kb)	Str.	N SNPs (N Attempted)
1	*SF3A3*	Splicing factor 3a, subunit 3, 60 kDa	10946	NM_006802.2	38,195,239	33.1	-	8 (8)
4	*MFSD7*	Major facilitator superfamily domain containing 7	84179	NM_032219.2	665,618	7.4	-	3 (3)
6	*ID4*	Inhibitor of DNA binding 4, dominant negative helix-loop-helix protein	3400	NM_001546.2	19,945,596	3.3	+	9 (9)
	*BTN3A3*	Butyrophilin, subfamily 3, member A3	10384	NM_006994.3	26,548,742	12.9	+	13 (13)
8	*OSGIN2*	Oxidative stress induced growth inhibitor family member 2	734	NM_004337.1	90,983,269	26.0	+	6 (7)
10	*HTRA1*	HtrA serine peptidase 1	5654	NM_002775.3	124,211,047	53.4	+	27 (27)
15	*C15orf15*	Chromosome 15 open reading frame 15	51187	NM_016304.2	53,260,804	15.7	-	15 (15)
16	*ZNF200*	Zinc finger protein 200	7752	NM_198087.1	3,212,343	13.1	-	5 (5)
17	*MSL1*	Male-specific lethal-1 homolog (Drosophila)	339287	NM_001012241.1	35,532,278	14.4	+	2 (2)
	*HEXIM1*	Hexamethylene bis-acetamide inducible 1	10614	NM_006460.2	40,580,467	4.8	+	3 (3)
19	*PTPRS*	Protein tyrosine phosphatase, receptor type, S	5802	NM_002850.3	5,109,506	182.3	-	28 (28)
	*CC2D1A*	Coiled-coil and C2 domain containing 1A	54862	NM_017721.3	13,878,052	24.6	+	7 (8)
	*PRPF31*	PRP31 pre-mRNA processing factor 31 homolog (S. cerevisiae)	26121	NM_015629.2	59,310,649	16.3	+	8 (8)
							**Total**	**134 (136)**

All genes are protein-coding and from UCSC (April 8, 2007) although *MSL1* is from NCBI (September 6, 2007); names for several genes changed from previous citations: *MFSD7* was *FLJ22269*, *OSGIN2* was *C8orf1*, *MSL1* was *LOC339287*, *HEXIM1* was *HIS1*, and *CC2DIA* was *FLJ20241*; N SNPs refers to number of tagSNPs or putative-functional SNPs analyzed; *FARP1* and *PRKCH* were excluded due to large gene size and low LD requiring over 100 tagSNPs each.

## Results

Demographic, reproductive, lifestyle, and tumor characteristics of 749 epithelial invasive ovarian cancer patients and 1,041 controls are described in Table 2. Generally, the expected distributions of risk factors were observed; a greater proportion of patients than controls had never used oral contraceptives (p<0.001), had used hormone therapy (p<0.001), were nulliparous (p = 0.01), and had a first or second degree family history of ovarian cancer (p<0.001). Such factors associated with risk of ovarian cancer were included as covariates in all genetic analyses.

**Table 2:** Characteristics of study participants.

		Patients (N = 749)	Controls (N = 1,041)	P-value
Age, years	Mean (S.D.)	58.4 (11.8)	57 (12.8)	**0.02**
Self-reported race	White	673 (91%)	944 (91%)	0.72
	African American	54 (7%)	79 (8%)	
	Asian	6 (1%)	5 (<1%)	
	Other	9 (1%)	9 (<1%)	
	Missing	7	4	
Body mass index, kg/m^2^	<23	173 (24%)	251 (25%)	0.17
	23–26	169 (23%)	247 (25%)	
	26–29	166 (23%)	247 (25%)	
	>29	216 (30%)	248 (25%)	
	Missing	25	48	
Oral contraceptive use	Never	306 (42%)	346 (35%)	**<0.001**
	1–48 Months	208 (29%)	253 (25%)	
	48+ Months	209 (29%)	400 (40%)	
	Missing	26	42	
Hormone therapy	Never	344 (49%)	588 (61%)	**<0.001**
	1–60 Months	195 (28%)	185 (19%)	
	60+ Months	169 (24%)	190 (20%)	
	Missing	41	78	
N children/age at first birth	Nulliparous	140 (19%)	141 (14%)	**0.01**
	1−2/< = 20 yrs	76 (1%)	97 (10%)	
	1−2/>20 yrs	231 (31%)	365 (36%)	
	3+/< = 20 yrs	134 (18%)	155 (15%)	
	3+/>20 yrs	158 (2%)	254 (25%)	
	Missing	10	29	
1^st^ or 2^nd^ degree family	Yes	85 (12%)	64 (6%)	**<0.001**
history of ovarian cancer	No	655 (89%)	952 (94%)	
	Missing	9	25	
Histologic sub-type	Serous	452 (61%)	-	-
	Endometrioid	126 (17%)	-	
	Clear cell	55 (7%)		
	Mucinous	36 (5%)	-	
	Other	77 (10%)	-	
	Missing	3	-	
Stage	I	150 (20%)	-	
	II	56 (8%)	-	-
	III	452 (62%)	-	
	IV	76 (10%)	-	
	Missing	15		

Data are counts (percentage) unless otherwise indicated. P-values are from t-test for continuous variables and Chi square test for categorical variables.

Gene-level and selected SNP-level association-testing results are shown in Table 3 and Table 4, respectively. Associations for the full set of SNPs examined are displayed in Table S1. Of genes examined in relation to risk of invasive ovarian cancer and risk of invasive serous ovarian cancer, only global variation in the *MSL1* and *PRPF31* genes was associated at p<0.05. *MSL1* gene-level principal components (summarized combinations of genotypes based on two SNPs) were associated with risk of invasive serous disease (p = 0.03). At one of the two SNPs in this gene, rs7211440 (r^2^ = 0.53 with rs17678694; Figure S1), carriage of the minor allele was associated with reduced risk of both invasive and invasive serous disease (OR 0.85, 95% CI 0.72–1.00, p = 0.05; OR 0.81, 95% CI 0.66–0.98, p = 0.03, respectively, Table 4), suggesting this SNP as the primary driver of *MSL1*'s gene-level serous association.

**Table 3:** Gene-level results.

					Invasive Disease (N = 749)		Invasive Serous Disease (N = 452)	
Chr.	Gene	N SNPs	N PC	N Haplotypes	PCp-value	Haplotypep-value	PCp-value	Haplotypep-value
1	*SF3A3*	8	3	55	0.74	0.80	0.53	0.16
4	*MFSD7*	3	3	7	0.19	0.51	0.19	0.73
6	*ID4*	9	4	86	0.30	0.52	0.24	0.69
	*BTN3A3*	13	5	78	0.63	0.81	0.32	0.54
8	*OSGIN2*	6	3	19	0.90	0.30	0.92	0.49
10	*HTRA1*	27	9	27	0.84	0.78	0.90	0.18
15	*C15orf15*	15	4	65	0.24	0.47	0.37	0.46
16	*ZNF200*	5	3	16	0.10	0.42	0.13	0.36
17	*MSL1*	2	2	3	0.16	0.25	**0.03**	0.23
	*HEXIM1*	3	3	5	0.30	0.45	0.24	0.14
19	*PTPRS*	28	13	26	0.52	0.73	0.46	0.95
	*CC2D1A*	7	4	30	0.21	0.77	0.19	0.80
	*PRPF31*	8	4	71	0.19	**0.03**	0.11	0.32

Analyses adjust for possible population structure (via principal components), study site, age, body mass index, hormone therapy, oral contraceptive use, number of live births, age at first live birth, and geographic region; N PC represents the number of principal components included in analysis of invasive cases; N haplotypes represents the estimated number of haplotypes with ten or more occurrences in the controls and invasive cases.

**Table 4:** Selected SNPs and risk of ovarian cancer.

Chr.	Gene	rsid	bp to previous	Invasive Disease (N = 749)		Serous Invasive Disease (N = 452)	
				per-allele OR (95% CI)	p-value	per-allele OR (95% CI)	p-value
4	*MFSD7*	rs7690350	na	0.87 (0.66–1.16)	0.34	0.95 (0.69–1.32)	0.76
		rs6840253	5,720	**0.81 (0.66–1.00)**	**0.05**	**0.76 (0.58–0.98)**	**0.03**
		rs4690290	2,939	1.03 (0.89–1.18)	0.70	1.03 (0.87–1.21)	0.74
6	*BTN3A3*	rs4711110	na	0.91 (0.74–1.12)	0.39	0.80 (0.62–1.02)	0.08
		rs12206812	779	0.81 (0.60–1.08)	0.15	**0.63 (0.44–0.90)**	**0.01**
		rs10456330	100	1.17 (0.92–1.48)	0.20	1.26 (0.96–1.66)	0.10
		rs12208390	414	1.15 (0.91–1.46)	0.24	1.24 (0.94–1.63)	0.12
		rs9379874	3,323	0.99 (0.86–1.14)	0.91	0.94 (0.79–1.11)	0.44
		rs17539219	113	0.98 (0.82–1.18)	0.86	0.89 (0.72–1.11)	0.32
		rs12214444	110	0.91 (0.74–1.12)	0.36	0.79 (0.62–1.02)	0.07
		rs13220495	988	1.00 (0.79–1.27)	0.99	0.88 (0.66–1.18)	0.39
		rs9379875	3,092	0.93 (0.75–1.15)	0.50	0.86 (0.66–1.12)	0.27
		rs3846845	183	1.01 (0.88–1.16)	0.90	0.95 (0.81–1.12)	0.56
		rs1796524	4,304	1.16 (0.92–1.46)	0.21	1.11 (0.84–1.47)	0.45
		rs6456723	6,286	0.92 (0.77–1.08)	0.31	0.89 (0.73–1.09)	0.26
		rs2273193	3,020	0.96 (0.82–1.12)	0.58	0.98 (0.82–1.18)	0.86
16	*ZNF200*	rs12917706	na	0.92 (0.80–1.07)	0.28	0.93 (0.79–1.10)	0.39
		rs401298	3,096	1.03 (0.90–1.20)	0.64	1.05 (0.88–1.24)	0.59
		rs9927763	1,301	1.06 (0.81–1.39)	0.66	1.16 (0.85–1.59)	0.35
		rs2075852	330	0.97 (0.85–1.12)	0.71	0.95 (0.80–1.12)	0.53
		rs186493	6,726	**0.83 (0.71–0.97)**	**0.02**	**0.82 (0.68–0.98)**	**0.03**
17	*MSL1*	rs17678694	na	1.01 (0.68–1.48)	0.97	0.72 (0.43–1.19)	0.20
		rs7211770	1,933	**0.85 (0.72–1.00)**	**0.05**	**0.81 (0.66–0.98)**	**0.03**
	*HEXIM1*	rs1053578	>5.0 Mb	1.28 (0.97–1.69)	0.08	**1.40 (1.02–1.91)**	**0.04**
		rs8070447	8,014	1.05 (0.85–1.29)	0.67	1.02 (0.80–1.30)	0.89
		rs7217422	1,166	1.03 (0.84–1.25)	0.79	0.98 (0.77–1.23)	0.83
19	*PTPRS*	rs8105746	na	0.97 (0.80–1.19)	0.80	1.05 (0.82–1.33)	0.72
		rs1143700	5,919	1.05 (0.87–1.26)	0.61	1.00 (0.81–1.25)	0.98
		rs1978237	2,866	1.00 (0.85–1.17)	0.99	0.97 (0.80–1.17)	0.72
		rs2302224	981	1.12 (0.88–1.44)	0.35	1.03 (0.76–1.38)	0.86
		rs2230611	1,860	1.04 (0.83–1.30)	0.73	1.06 (0.81–1.39)	0.67
		rs11085118	1,676	1.03 (0.83–1.27)	0.81	1.01 (0.78–1.31)	0.95
		rs10413063	2,125	1.03 (0.90–1.19)	0.66	1.05 (0.89–1.24)	0.55
		rs12975955	3,520	0.95 (0.82–1.09)	0.47	0.98 (0.82–1.16)	0.79
		rs10412973	1,097	1.00 (0.84–1.20)	1.00	0.98 (0.79–1.21)	0.84
		rs12610082	6,108	1.15 (0.96–1.38)	0.13	1.15 (0.93–1.42)	0.19
		rs4807015	4,127	0.93 (0.81–1.07)	0.32	0.94 (0.80–1.11)	0.47
		rs2379609	5,031	1.14 (0.97–1.35)	0.11	1.15 (0.95–1.41)	0.15
		rs3746130	4,226	1.03 (0.82–1.29)	0.81	1.00 (0.76–1.31)	0.98
		rs10415488	6,325	1.02 (0.89–1.18)	0.75	1.04 (0.88–1.22)	0.68
		rs886936	6,715	0.91 (0.79–1.04)	0.18	**0.85 (0.72–1.00)**	**0.05**
		rs11878779	1,286	0.87 (0.75–1.01)	0.06	**0.79 (0.66–0.95)**	**0.01**
		rs17130	252	1.08 (0.93–1.25)	0.31	1.10 (0.93–1.31)	0.27
		rs4807016	3,520	1.00 (0.86–1.15)	0.99	0.98 (0.82–1.16)	0.78
		rs2238640	3,016	1.07 (0.93–1.25)	0.34	1.09 (0.91–1.30)	0.36
		rs1034863	6,245	0.93 (0.76–1.14)	0.47	0.96 (0.76–1.22)	0.75
		rs933394	1,280	0.85 (0.63–1.14)	0.27	0.80 (0.56–1.15)	0.23
		rs8110570	427	1.00 (0.82–1.23)	0.98	1.06 (0.83–1.34)	0.64
		rs1141371	4,113	0.97 (0.82–1.14)	0.70	0.95 (0.78–1.15)	0.61
		rs1034917	813	0.86 (0.70–1.06)	0.16	0.94 (0.74–1.19)	0.61
		rs7254570	4,494	1.23 (0.93–1.62)	0.14	1.24 (0.90–1.71)	0.18
		rs740058	9,304	0.90 (0.76–1.07)	0.23	0.94 (0.77–1.15)	0.55
		rs4807711	2,173	0.92 (0.75–1.12)	0.42	1.00 (0.79–1.26)	0.98
		rs758512	529	0.81 (0.60–1.09)	0.16	0.88 (0.63–1.23)	0.46
	*CC2D1A*	rs8111004	>8.6 Mb	1.07 (0.90–1.26)	0.45	1.08 (0.89–1.32)	0.43
		rs3745457	4,609	1.09 (0.92–1.28)	0.31	1.10 (0.90–1.34)	0.34
		rs6511901	10,878	1.09 (0.92–1.30)	0.32	1.13 (0.92–1.38)	0.24
		rs2305777	11,139	**0.84 (0.72–0.99)**	**0.03**	**0.76 (0.62–0.92)**	**0.005**
		rs1059721	2,810	1.14 (0.95–1.38)	0.17	1.10 (0.88–1.37)	0.40
		rs2305778	2,686	0.89 (0.67–1.20)	0.45	0.97 (0.70–1.36)	0.87
		rs2305779	746	0.97 (0.78–1.21)	0.78	0.93 (0.71–1.21)	0.59
	*PRPF31*	rs4806711	>45 Mb	1.00 (0.84–1.20)	0.97	0.99 (0.80–1.22)	0.89
		rs12985735	4,358	1.13 (0.98–1.30)	0.10	1.12 (0.94–1.33)	0.19
		rs11670086	4,076	1.04 (0.84–1.29)	0.72	1.14 (0.89–1.47)	0.30
		rs254272	2,072	1.05 (0.89–1.25)	0.56	1.08 (0.88–1.32)	0.47
		rs10424816	511	1.03 (0.89–1.19)	0.71	1.05 (0.89–1.25)	0.55
		rs254271	549	1.06 (0.92–1.23)	0.41	1.05 (0.89–1.25)	0.56
		rs8102427	391	1.04 (0.90–1.20)	0.62	1.03 (0.87–1.23)	0.70
		rs4806716	8,720	1.07 (0.90–1.26)	0.45	1.16 (0.95–1.41)	0.14

Results are shown for all SNPs in genes with a gene- or SNP-level p-value<0.05; bp to previous represents distance in base pairs between SNPs; odds ratios, 95% confidence intervals and p-values from logistic regression analysis, adjusted for possible population structure (via principal components), study site, age, body mass index, hormone therapy, oral contraceptive use, number of live births, age at first live birth, and geographic region; per-allele odds ratios and tests for trend p-values based on an ordinal (log-additive) genotypic response; bold indicates p-value<0.05.

*PRPF31* haplotypes (consecutive series of alleles based on eight SNPs) were associated with risk of invasive disease (p = 0.03). As haplotype analysis can reveal hidden associations, we more closely examined the twenty-six common haplotypes within *PRPF31* (Table S2). Compared to the most common haplotype 11111111 (major alleles at all *PRPF31* SNPs), the haplotype 10101111 (minor alleles at intronic tagging single nucleotide polymorphisms (tagSNPs) rs12985735 and rs254272) conferred reduced risk of invasive ovarian cancer (OR 0.22, 95%CI 0.06–0.82, p = 0.02). These two SNPs were only modestly correlated (r^2^ = 0.23; Figure S1), and neither was independently associated with risk (rs12985735 per allele OR 1.13, 95% CI 0.98–1.30, p = 0.10; rs254272 per allele OR 1.05, 95% CI 0.89–1.25, p = 0.56), suggesting that an ungenotyped variant in close proximity to the *PRPF31* haplotype may contribute to the association. As two other *PRPF31* haplotypes were associated with ovarian cancer risk at p<0.10 (00111101 OR 2.81, 95% CI 0.85–9.22, p = 0.09; 10101101 OR 2.86, 95% CI 0.94–8.71, p = 0.06), other variants in the gene may also contribute to the observed global haplotype association.

Outside of the two gene-level associated genes (*MSL1* and *PRPF31*), a small number of SNPs in six other genes were associated at the single-SNP level (p<0.05, Table 4) including three SNPs associated with reduced risk of both invasive and invasive serous disease (in *CC2DIA*, *MFSD7*, and *ZNF200*). The strongest SNP-level association was observed at the non-synonymous rs2305777 (T801M) in the NF-κB activating gene *CC2D1A* (invasive OR 0.84, 95% 0.72–0.99, p = 0.03; serous invasive OR 0.76, 95% CI 0.62–0.99 p = 0.005). Based on sequence conservation across species [14], this SNP is predicted to be relatively undamaging to protein function. Because this SNP was not correlated with other genotyped SNPs (r^2^<0.12, Figure S1) and did not tag other common HapMap SNPs at r^2^>0.9, sequencing may be required to clarify the meaning of this SNP association. Similarly, *MFSD7* rs6840253 was associated with a reduction of ovarian cancer risk (invasive OR 0.81, 95% CI 0.66–1.0, p = 0.05; serous invasive OR 0.76, 95% CI 0.58–0.98, p = 0.03), as was *ZNF200* rs186493 (invasive OR 0.83, 95% 0.71–0.82, p = 0.02; invasive serous OR 0.82, 95% CI 0.68–0.98, p = 0.03). Both are promoter region SNPs (within 5 kb 5′ upstream) and independent of other HapMap SNPs at r^2^>0.9. Because each is correlated at r^2^>0.6 with other genotyped SNPs, additional genotyping of modestly correlated SNPs could help elucidate these associations.

Three SNPs were associated only with reduced risk of invasive serous disease (in *PTPRS* and *BTN3A3*), and one SNP associated with increased risk of invasive serous disease (in *HEXIM1*). Thus, while results were generally similar in both case groups, the often greater statistical significance of results among women with serous disease, despite reduced power due to a 40% smaller sample size, suggests that subtype analysis revealed heterogeneity by histology.

SNPs that were suggestive only in serous disease included two highly-correlated *PTPRS* intron 9 SNPs (r^2^ = 0.99; rs886936 OR 0.85, 95% CI 0.72–1.00, p = 0.05; rs11878779 OR 0.79, 95% CI 0.66–0.95, p = 0.01). Interestingly, these and three other highly-correlated intron 9 SNPs (Figure S1) were independent of each other in HapMap at r^2^<0.9, serving as an example of non-transferability of linkage disequilibrium (LD) across populations. The only SNP with minor alleles associated with increased risk was the potential *HEXIM1* promoter region SNP rs1053578 (serous OR 1.40, 95% CI 1.02–1.91, p = 0.04) which was independent of other genotyped SNPs at r^2^<0.04 and of other HapMap SNPs at r^2^<0.9. *BTN3A3*'s putative promoter region SNP rs12206812 was associated with reduced risk of serous invasive disease (OR 0.63, 95% CI 0.44–0.90, p = 0.01); however, we suspect genotype or genetic map error because of failure in WGA DNA and independence with all other genotyped *BTN3A3* SNPs (r^2^ = 0; Figure S1). No gene-level or SNP-level associations at p<0.05 were observed for *SF3A3*, *ID4*, *OSGIN2*, *HTRA1*, or *C15orf15*.

## Discussion

Knowledge about the genetics of ovarian cancer is in a rapid state of expansion. As the most lethal gynecologic cancer, discovery of inherited factors related to etiology and outcome may assist in the development of important targeted prediction and therapeutic strategies. Recent work has clarified the roles of long-standing candidate SNPs in the progesterone receptor, retinoblastoma, p53, and cell cycle genes [10], and enabled genome-wide association studies [8]. Analysis of highly-informative variants in selective sets of novel genes complements these candidate SNP and genome-wide association studies, providing improved coverage in high-priority regions based on known tumor biology [16]. Here, we selected novel candidate genes based on prior evidence of their association with time to relapse of ovarian cancer, and we chose comprehensive sets of variants [2], [13]. Our primary result is that variation in *MSL1* was related to risk of serous invasive ovarian cancer; notably, minor alleles at rs7211440 correlated with decreased risk (OR 0.81, 95% CI 0.66–0.98, p = 0.03). Previously, increased expression of MSL1 was correlated with earlier time to relapse [2]; additional validation of the prognostic model and replication of the etiologic association are warranted.

*MSL1* encodes one of five proteins that form the highly-conserved MSL complex with enzymatic capabilities as a histone acetyltransferase (HAT) [17]. HATs modify a variety of histone domains through acetylation, which, along with other coactivators, regulates histone and chromatin activation and influences gene expression [18]. The MSL complex specifically acetylates lysine residue 16 on histone H4 (H4-Lys16), which plays a crucial role in regulating chromatin folding and silencing of gene expression [19], [20], [17]. Knock-out models indicate that absence of the MSL complex leads to malfunctions during the S phase of the cell cycle, leading to errors in DNA replication [17]. Additionally, loss of monoacetylation of H4-Lys16 and aberrant functioning on H4 are hallmarks of cancer cells. Our data suggest that inherited variation in *MSL1* may impact risk of invasive serous ovarian cancer and are consistent with findings that irregular H4 modifications may cause errors in chromatin folding and gene expression and are widespread in cancer phenotypes [21]. With suggestive SNPs in genes for p53 and CDKN2A [10], [9], which also regulate histone modification, evidence for a role of inherited risk factors related to histones is accumulating.

Strengths of this work include the use of two case-control study populations (from Mayo Clinic and Duke University), advanced SNP selection techniques (e.g., high level of required correlation among alleles, inclusion of putative-functional SNPs, and selection of multiple tagSNPs in large LD bins), and excellent genotyping quality. Our assessment of risk for serous invasive disease suggested a degree of genetic heterogeneity by histologic subtype; however, we suggest caution in interpretation of results (particularly single-SNP results in the absence of gene-level significance) due to the relatively large number of tests performed. We also note that no results are statistically significant after adjustment for multiple testing using a conservative Bonferroni correction. Thus, replication of our results is warranted to confirm these associations. Avenues for future research extending from this expression-based candidate gene work include analysis of other histone regulatory genes and detailed assessment of functional mechanisms. More imminently, examination of promising SNPs within *MSL1* among a larger set of serous invasive patients and controls and additional fine-scale mapping [16] will assist in clarification of the importance of these genes to ovarian cancer susceptibility. This should, in turn, inform translation of such findings to the clinical management of women at increased risk for ovarian cancer.

## Materials and Methods

### Study Participants

Subjects participated in two ongoing case-control studies of epithelial ovarian cancer initiated in January 2000 at Mayo Clinic (Rochester, MN) and in May 1999 at Duke University (Durham, NC). Details of the study design have been described in more detail elsewhere [22]–[24]. Briefly, a total of 749 women with histologically-confirmed invasive epithelial ovarian cancer and 1,041 controls without ovarian cancer and without bilateral oophorectomy were recruited from the two study sites (Table 2; site-specific characteristics provided in Table S3). At Mayo Clinic, ovarian cancer cases (patients) were over 20 years of age with histologically confirmed incident epithelial ovarian cancer and enrolled in the study within one year after diagnosis. All cases seen in the gynecologic or medical oncology units which lived in the six-state region that defines the primary service population of Mayo Clinic (Minnesota, Iowa, Wisconsin, Illinois, North Dakota, and South Dakota) were invited to participate. Controls were recruited from among women seen for general medical examinations and frequency-matched to patients on age and region of residence (i.e., state, county). At Duke University, patients were women with histologically confirmed primary epithelial ovarian cancer, between 20 and 74 years of age, and identified within a 48-county region using the North Carolina Central Cancer Registry. Controls were identified using list-assisted random digit dialing and frequency matched to patients on race, age, and county of residence. No exclusions based on ethnicity were made. Applicants provided written informed consent, and protocols were approved by the Mayo Clinic and Duke University Institutional Review Board.

### Data and Biospecimen Collection

Information on potential risk factors was collected through in-person interviews at both sites using similar questionnaires. DNA was extracted from 10 to 15 mL fresh venous blood using the Gentra AutoPure LS Purgene salting out methodology (Gentra, Minneapolis, MN). DNA from Duke University participants were transferred to Mayo Clinic for whole-genome amplification (WGA) with the REPLI-G protocol (Qiagen Inc, Valencia CA) which we have shown yielded highly-reproducible results [25]. DNA concentrations were adjusted to 50 ng/µl prior to genotyping and verified using the PicoGreen dsDNA Quantitation kit (Molecular Probes, Inc., Eugene OR). Samples were bar-coded to ensure accurate and reliable processing.

### SNP Selection

We identified tagSNPs within five kb of each candidate gene using the algorithm of ldSelect [26] to bin pair-wise correlated SNPs at r^2^≥0.90 with minor allele frequency (MAF) ≥0.05 in the HapMap CEU population (Utah residents with ancestry from northern and western Europe) [27]. HapMap data were used because, in November 2007, they were more informative for these genes than data from Perlegen Sciences [28], Seattle SNPs (http://pga.mbt.washington.edu), and NIEHS SNPs (http://www.egp.gs.washingon.edu). One tagSNP per bin was selected if less than ten SNPs were in a LD bin, and two tagSNPs per bin were selected in LD bins with ten or more SNPs. Among tagSNPs, SNPs were chosen to maximize Illumina SNP score (a measure of predicted genotyping success) and then MAF. *FARP1* (307 kb) and *PRKCH* (229 kb) required over 100 tagSNPs each and were excluded from the study for cost-efficiency. For the remaining genes (Table 1), 117 tagSNPs and 19 putative-functional SNPs (within 10 kb upstream, 5′ UTR, 3′ UTR, or non-synonymous from Ensembl version 34 with European-American MAF≥0.05 and Illumina SNP score≥0.6) were selected. Thus, a total of 136 SNPs in these 13 candidate genes were genotyped.

### Genotyping

As part of a larger study, genotyping of 2,176 DNA samples (897 genomic, 1,279 WGA, and 129 duplicates) from 2,047 unique study participants was performed at Mayo Clinic along with 65 laboratory controls. We used the Illumina GoldenGate BeadArray assay and BeadStudio software for automated clustering and calling according to a standard protocol [29]. Of 2,047 participants genotyped, 44 samples (2.1%) failed (call rate <90%), and 213 participants (10.4%) were found to be ineligible or have borderline disease and were excluded; thus 1,790 participants (including 749 patients with invasive disease and 1,041 controls) were analyzed here. A total of 1,152 SNPs for a variety of projects were attempted; 25 failed SNPs included 15 (1.3%) with call rate <90%, nine (0.8%) with poor clustering, and one (<0.1%) with unresolved replicate or Mendelian errors in genomic DNA. We assessed departures from Hardy Weinberg equilibrium (HWE) in self-reported white, non-Hispanic controls with a Pearson goodness-of-fit test or, in the case of SNPs with a MAF <5%, a Fisher exact test, and we excluded SNPs with MAF <0.01 (N = 64, 5.6%) or HWE p-value<0.0001 (N = 11, 1.0%), leaving 1,052 SNPs for analysis. For WGA DNA, an additional 20 SNPs (1.7%) were excluded due to one or more of the above criteria. Among the 13 candidate genes studied, 134 of 136 SNPs were successfully genotyped in genomic samples, and all but six of these were also genotyped successfully in WGA samples (Table S4).

### Statistical Methods

Distributions of demographic and clinical variables were compared between patients and controls using chi-square tests or t-tests, and estimates of pair-wise LD between SNPs were obtained using Haploview software, version 4.1 [30]. Genetic association analyses (described below) were adjusted for study site, age, body mass index, hormone therapy, oral contraceptive use, number of live births, age at first live birth, and population structure principal components which accounted for the possibility of population stratification using an approach similar to that described previously [31]. Population structure principal components were created using 2,517 SNPs from this and prior genotyping panels [23]; scatter plot matrices by self-reported race indicated that the first four population structure principal components reasonably approximated racial differences across individuals and were thus included as covariates in all models (Figure S2).

Associations with ovarian cancer risk were assessed using logistic regression of SNPs, gene-level principal components, and gene-level haplotypes. For SNPs, odds ratios (OR) and 95% confidence intervals (CI) were estimated separately for heterozygous and homozygous minor allele genotypes, using the homozygous major allele genotype as the referent group. We included eight SNPs with HWE<0.05 (Table S4) because of acceptable genotype cluster plots, the large number of tests (Bonferroni corrected p-value≤3.7×10^−4^), and no assumption of HWE for single-SNP analysis. Formal genotypic tests of association were carried out assuming an ordinal (log-additive) effect using simple tests for trend. Within each gene, we used a principal component analysis to create orthogonal linear combinations of the SNP minor allele count variables (including genotypes imputed using the MACH software package [32]) to provide an alternate and equivalent representation of the collection of SNPs as a whole. The resulting smallest subset of gene-level principal components that accounted for at least 90% of the SNP variability was included in regression models, and gene-specific associations were evaluated using a multiple degree of freedom likelihood ratio test. Gene-centric haplotype-based association analyses were conducted using posterior probabilities of all possible haplotypes for an individual (excluding SNPs with HWE p-value<0.05), conditional on the observed genotypes. The expectation-maximization algorithm was used to estimate haplotypes [33] and create haplotype design variables ranging from 0 to 2. Because of the imprecision involved in low-frequency haplotypes, we excluded haplotypes with an estimated frequency of less than ten. Assessments of risk among common haplotypes tested the simultaneous effects of all haplotypes combined in logistic regression; individual haplotype associations used the most common haplotype as reference. All statistical tests were two-sided, and unless otherwise indicated, were carried out using SAS software (SAS Institute, Inc., Cary, NC).

## Supporting Information

Figure S1 Linkage disequilibrium plots. Genes with gene-level or SNP-level p<0.05 are shown; Haploview 4.1 (Barrett et al., 2005) based on self-reported white-non-Hispanic controls; r2 = 0 = white and r2 = 1 = black; numbers represent r2 * 100.
(1.04 MB DOC)

Figure S2 Matrix of scatterplots for four population structure principal components by self-reported race. Population structure principal components analysis based on 1,981 participants and 2,517 SNPs including imputed genotypes; for each scatterplot, vertical axis corresponds to the component listed in diagonal element to the left of the plot, and horizontal axis corresponds to the component listed in diagonal underneath the plot; results suggest that the first component differentiated white non-Hispanic and black non-Hispanic from other samples, while the fourth component helped to further differentiate Asian from other samples; these four population structure principal components were used as covariates in association testing.
(0.30 MB DOC)

Table S1 SNPs and risk of ovarian cancer, OR (95% CI)
(0.43 MB DOC)

Table S2 Haplotype results for PRPF31 (global p-value = 0.03)
(0.12 MB DOC)

Table S3 Characteristics of study participants by study site
(0.14 MB DOC)

Table S4 SNP and genotype information
(0.56 MB DOC)
