# Supplementary figures and images for: Risk of Ovarian Cancer and Inherited Variants in Relapse-Associated Genes
Source: PLoS One. 2010 Jan 27;5(1):e8884. doi: 10.1371/journal.pone.0008884 (PMC2811736; doi:10.1371/journal.pone.0008884)

***Figure S1. Linkage disequilibrium plots***

| *ZNF200*  *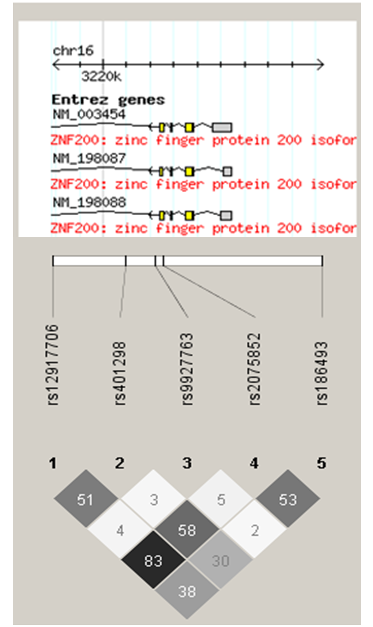* | *MSL1*  *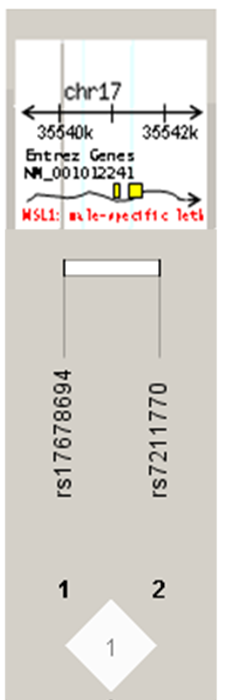* | *HEXIM1*  *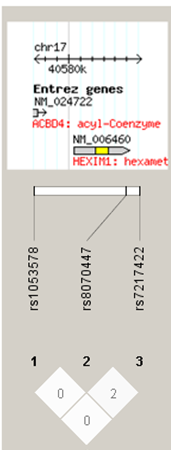* | | *MFSD7*  *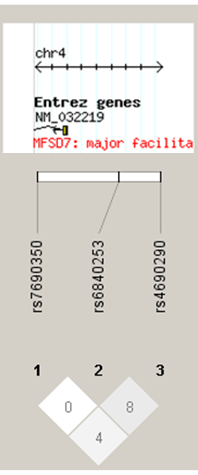* | *PRPF31*  *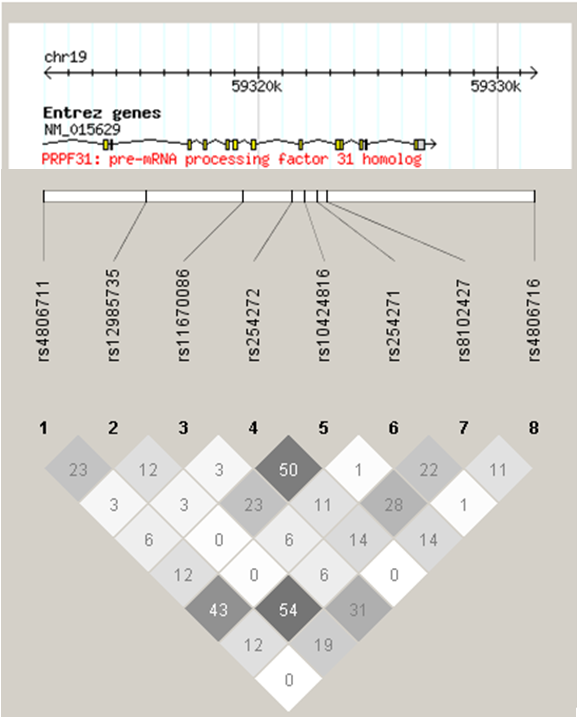* |
| --- | --- | --- | --- | --- | --- |
| *CC2D1A*  *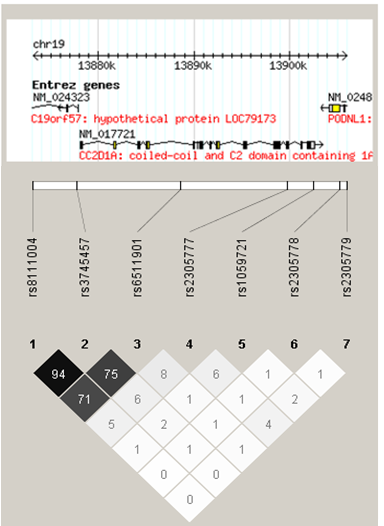* | | | *BTN3A3*  *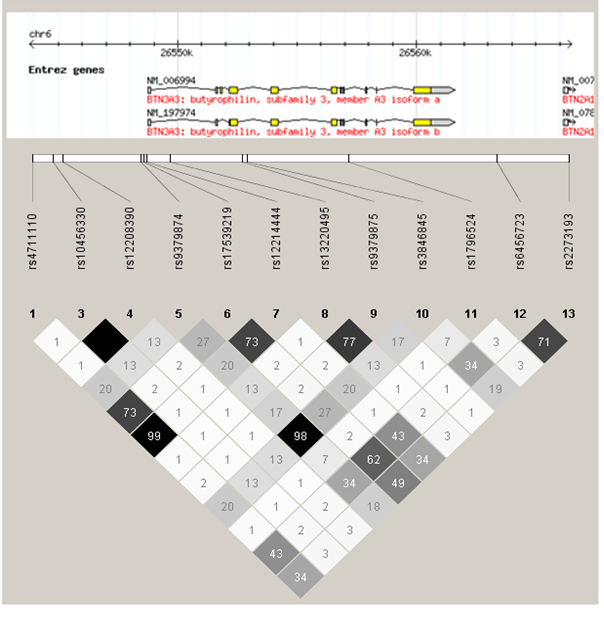* | | |
| *PTPRS*  *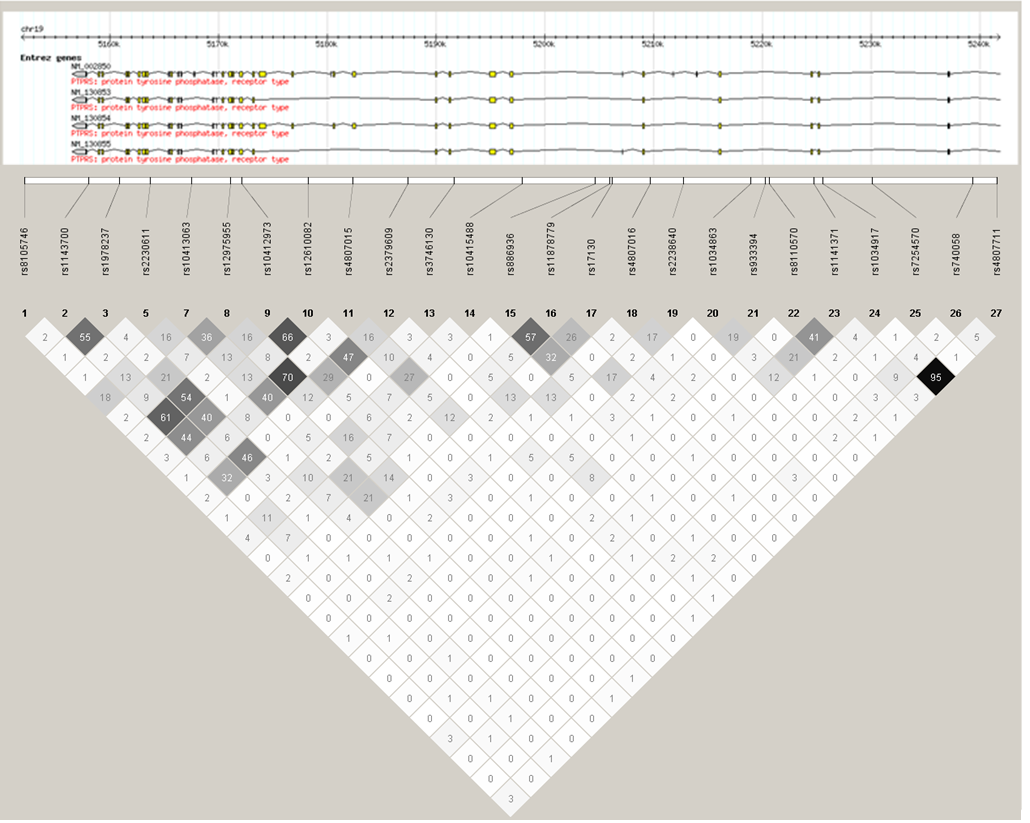* | | | | | |

Supplement: Figure S1 — Linkage disequilibrium plots. Genes with gene-level or SNP-level p<0.05 are shown; Haploview 4.1 (Barrett et al., 2005) based on self-reported white-non-Hispanic controls; r2 = 0 = white and r2 = 1 = black; numbers represent r2 * 100. (1.04 MB DOC) [file pone.0008884.s001.doc]
