# Supplementary material for: Risk of Ovarian Cancer and Inherited Variants in Relapse-Associated Genes
Source: PLoS One. 2010 Jan 27;5(1):e8884. doi: 10.1371/journal.pone.0008884 (PMC2811736; doi:10.1371/journal.pone.0008884)

**Figure S2. Matrix of scatterplots for four population structure principal components by self-reported race**

**
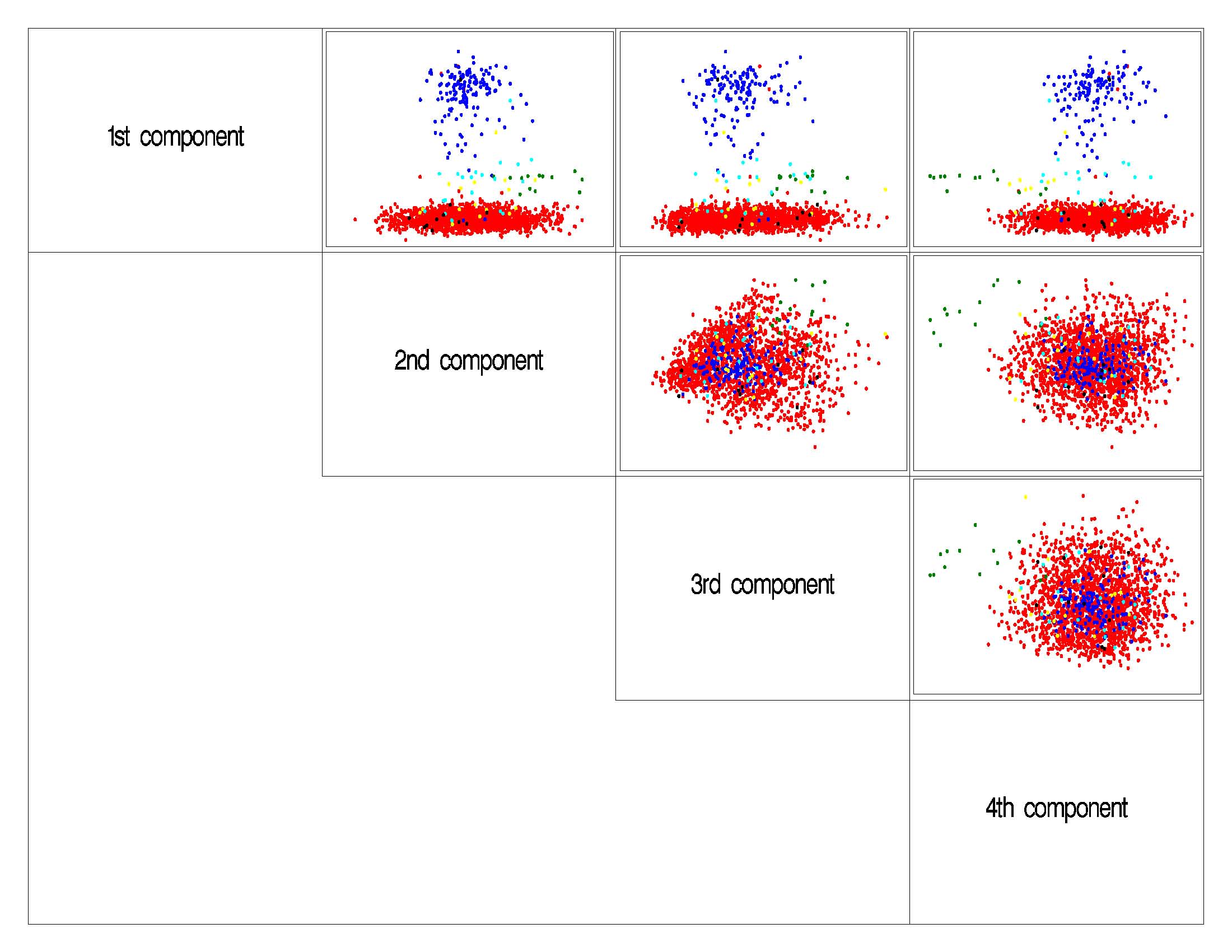
**


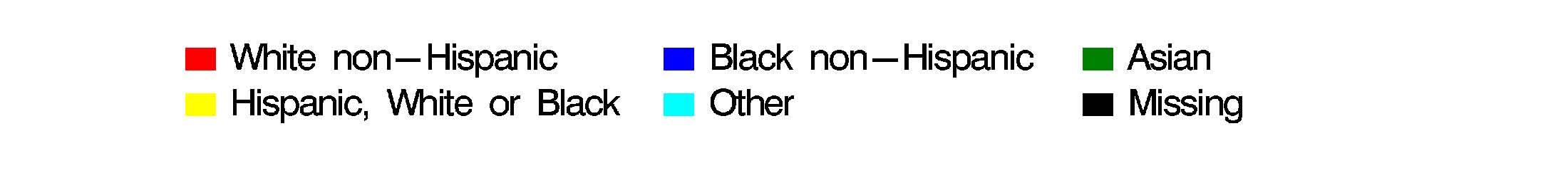

Supplement: Figure S2 — Matrix of scatterplots for four population structure principal components by self-reported race. Population structure principal components analysis based on 1,981 participants and 2,517 SNPs including imputed genotypes; for each scatterplot, vertical axis corresponds to the component listed in diagonal element to the left of the plot, and horizontal axis corresponds to the component listed in diagonal underneath the plot; results suggest that the first component differentiated white non-Hispanic and black non-Hispanic from other samples, while the fourth component helped to further differentiate Asian from other samples; these four population structure principal components were used as covariates in association testing. (0.30 MB DOC) [file pone.0008884.s002.doc]
