# Supplementary material for: Risk of Ovarian Cancer and Inherited Variants in Relapse-Associated Genes
Source: PLoS One. 2010 Jan 27;5(1):e8884. doi: 10.1371/journal.pone.0008884 (PMC2811736; doi:10.1371/journal.pone.0008884)
Supplement: Table S1 — SNPs and risk of ovarian cancer, OR (95% CI) (0.43 MB DOC) [file pone.0008884.s003.doc]

**~~Table S1. SNPs and risk of ovarian cancer, OR (95% CI)~~**

|  |  |  | **bp to previous** | **Invasive Disease** | | | | **Serous Invasive Disease** | | | |
| --- | --- | --- | --- | --- | --- | --- | --- | --- | --- | --- | --- |
| **Chr.** | **Gene** | **rsid** |  | **AB v AA** | **BB v AA** | **per-allele** | **p-trend** | **AB v AA** | **BB v AA** | **per-allele** | **p-trend** |
| 1 | *SF3A3* | rs17465651 | na | 0.92 (0.74-1.16) | 1.03 (0.77-1.38) | 1.00 (0.87-1.16) | 0.96 | 0.83 (0.64-1.08) | 0.96 (0.69-1.36) | 0.96 (0.81-1.14) | 0.63 |
|  |  | rs17465826 | 5,025 | 0.96 (0.66-1.39) | 1.15 (0.06-23.4) | 0.96 (0.67-1.38) | 0.85 | 0.87 (0.56-1.36) | - | 0.86 (0.56-1.33) | 0.50 |
|  |  | rs1050257 | 67 | 0.88 (0.69-1.12) | 0.66 (0.29-1.52) | 0.86 (0.70-1.07) | 0.18 | 0.91 (0.69-1.21) | 0.38 (0.11-1.35) | 0.85 (0.66-1.10) | 0.22 |
|  |  | rs9293 | 265 | 1.00 (0.70-1.42) | 0.42 (0.08-2.16) | 0.93 (0.67-1.29) | 0.65 | 1.00 (0.66-1.51) | - | 0.88 (0.60-1.30) | 0.52 |
|  |  | rs6660034 | 20,480 | 0.91 (0.72-1.16) | 0.76 (0.34-1.71) | 0.90 (0.73-1.12) | 0.35 | 0.97 (0.73-1.28) | 0.54 (0.18-1.66) | 0.91 (0.71-1.18) | 0.49 |
|  |  | rs7512424 | 2,933 | 0.92 (0.72-1.17) | 0.77 (0.34-1.71) | 0.91 (0.73-1.12) | 0.38 | 0.97 (0.73-1.28) | 0.54 (0.18-1.66) | 0.91 (0.71-1.18) | 0.49 |
|  |  | rs7528185 | 4,793 | 0.91 (0.74-1.11) | 1.57 (1.07-2.31) | 1.08 (0.92-1.26) | 0.33 | 0.93 (0.73-1.19) | 1.84 (1.19-2.85) | 1.15 (0.96-1.38) | 0.13 |
|  |  | rs4072980 | 4,047 | 0.91 (0.73-1.13) | 1.12 (0.84-1.49) | 1.03 (0.90-1.19) | 0.63 | 0.85 (0.66-1.11) | 1.18 (0.85-1.64) | 1.05 (0.89-1.24) | 0.56 |
| 4 | *MFSD7* | rs7690350 | na | 0.91 (0.65-1.26) | 0.66 (0.27-1.61) | 0.87 (0.66-1.16) | 0.34 | 0.97 (0.66-1.44) | 0.83 (0.29-2.34) | 0.95 (0.69-1.32) | 0.76 |
|  |  | rs6840253 | 5,720 | 0.82 (0.64-1.05) | 0.62 (0.30-1.30) | **0.81 (0.66-1.00)** | **0.05** | 0.79 (0.59-1.06) | 0.42 (0.15-1.17) | **0.76 (0.58-0.98)** | **0.03** |
|  |  | rs4690290 | 2,939 | 0.98 (0.77-1.23) | 1.06 (0.80-1.40) | 1.03 (0.89-1.18) | 0.70 | 1.04 (0.79-1.38) | 1.06 (0.76-1.47) | 1.03 (0.87-1.21) | 0.74 |
| 6 | *ID4* | rs9348399 | na | 1.05 (0.84-1.32) | 0.70 (0.32-1.56) | 1.00 (0.82-1.22) | 0.99 | 0.98 (0.75-1.28) | 1.05 (0.46-2.43) | 0.99 (0.79-1.26) | 0.96 |
|  |  | rs6926229 | 308 | 1.03 (0.82-1.29) | 1.04 (0.77-1.39) | 1.02 (0.88-1.18) | 0.79 | 0.91 (0.70-1.18) | 1.01 (0.72-1.41) | 0.99 (0.84-1.17) | 0.90 |
|  |  | rs6456278 | 3,002 | 0.97 (0.76-1.23) | 1.06 (0.81-1.39) | 1.03 (0.90-1.18) | 0.67 | 0.96 (0.72-1.27) | 1.11 (0.81-1.53) | 1.06 (0.90-1.24) | 0.51 |
|  |  | rs6922714 | 345 | 0.98 (0.79-1.20) | 0.98 (0.71-1.35) | 0.98 (0.85-1.14) | 0.84 | 0.94 (0.73-1.20) | 0.95 (0.65-1.39) | 0.96 (0.81-1.15) | 0.67 |
|  |  | rs12528525 | 603 | 0.87 (0.71-1.08) | 1.02 (0.71-1.45) | 0.95 (0.82-1.11) | 0.54 | 0.84 (0.66-1.08) | 0.96 (0.63-1.46) | 0.92 (0.77-1.11) | 0.39 |
|  |  | rs1980461 | 3,191 | 0.97 (0.76-1.23) | 1.08 (0.82-1.42) | 1.04 (0.91-1.19) | 0.59 | 0.91 (0.68-1.20) | 1.03 (0.75-1.41) | 1.01 (0.86-1.19) | 0.87 |
|  |  | rs8214 | 4,770 | 0.87 (0.70-1.08) | 0.67 (0.41-1.07) | 0.85 (0.71-1.01) | 0.06 | 0.80 (0.62-1.03) | 0.69 (0.40-1.20) | 0.81 (0.66-1.00) | 0.05 |
|  |  | rs1047033 | 713 | 1.13 (0.92-1.39) | 1.10 (0.78-1.56) | 1.08 (0.93-1.25) | 0.34 | 1.15 (0.90-1.47) | 1.19 (0.79-1.78) | 1.11 (0.93-1.33) | 0.24 |
|  |  | rs6906699 | 3,863 | 1.06 (0.86-1.30) | 0.93 (0.60-1.43) | 1.01 (0.86-1.19) | 0.90 | 1.11 (0.87-1.42) | 1.06 (0.64-1.76) | 1.07 (0.89-1.30) | 0.47 |
|  |  | rs6456723 | 6,286 | 0.93 (0.75-1.15) | 0.81 (0.53-1.24) | 0.92 (0.77-1.08) | 0.31 | 0.85 (0.66-1.10) | 0.87 (0.53-1.42) | 0.89 (0.73-1.09) | 0.26 |
|  |  | rs2273193 | 3,020 | 0.96 (0.78-1.18) | 0.91 (0.61-1.33) | 0.96 (0.82-1.12) | 0.58 | 0.92 (0.72-1.17) | 1.07 (0.69-1.66) | 0.98 (0.82-1.18) | 0.86 |
|  | *BTN3A3* | rs4711110 | > 6.9 Mb | 0.95 (0.75-1.20) | 0.69 (0.33-1.47) | 0.91 (0.74-1.12) | 0.39 | 0.80 (0.60-1.07) | 0.62 (0.25-1.58) | 0.80 (0.62-1.02) | 0.08 |
|  |  | rs12206812 | 779 | 0.86 (0.62-1.20) | 0.42 (0.13-1.35) | 0.81 (0.60-1.08) | 0.15 | 0.62 (0.41-0.93) | 0.44 (0.12-1.65) | **0.63 (0.44-0.90)** | **0.01** |
|  |  | rs10456330 | 100 | 1.16 (0.89-1.51) | 1.48 (0.56-3.87) | 1.17 (0.92-1.48) | 0.20 | 1.22 (0.90-1.67) | 1.90 (0.65-5.58) | 1.26 (0.96-1.66) | 0.10 |
|  |  | rs12208390 | 414 | 1.16 (0.89-1.51) | 1.30 (0.51-3.33) | 1.15 (0.91-1.46) | 0.24 | 1.23 (0.90-1.67) | 1.67 (0.58-4.78) | 1.24 (0.94-1.63) | 0.12 |
|  |  | rs9379874 | 3,323 | 0.81 (0.65-1.03) | 1.01 (0.77-1.34) | 0.99 (0.86-1.14) | 0.91 | 0.80 (0.61-1.05) | 0.90 (0.65-1.26) | 0.94 (0.79-1.11) | 0.44 |
|  |  | rs17539219 | 113 | 1.04 (0.84-1.29) | 0.75 (0.41-1.39) | 0.98 (0.82-1.18) | 0.86 | 0.91 (0.70-1.17) | 0.76 (0.38-1.55) | 0.89 (0.72-1.11) | 0.32 |
|  |  | rs12214444 | 110 | 0.95 (0.75-1.21) | 0.63 (0.29-1.37) | 0.91 (0.74-1.12) | 0.36 | 0.81 (0.61-1.08) | 0.53 (0.19-1.43) | 0.79 (0.62-1.02) | 0.07 |
|  |  | rs13220495 | 988 | 1.10 (0.84-1.43) | 0.51 (0.18-1.47) | 1.00 (0.79-1.27) | 0.99 | 0.99 (0.72-1.36) | 0.29 (0.06-1.33) | 0.88 (0.66-1.18) | 0.39 |
|  |  | rs9379875 | 3,092 | 0.96 (0.75-1.23) | 0.72 (0.32-1.63) | 0.93 (0.75-1.15) | 0.50 | 0.96 (0.72-1.29) | 0.32 (0.09-1.14) | 0.86 (0.66-1.12) | 0.27 |
|  |  | rs3846845 | 183 | 0.83 (0.66-1.04) | 1.05 (0.79-1.39) | 1.01 (0.88-1.16) | 0.90 | 0.81 (0.62-1.06) | 0.93 (0.67-1.30) | 0.95 (0.81-1.12) | 0.56 |
|  |  | rs1796524 | 4,304 | 1.12 (0.87-1.45) | 1.74 (0.64-4.74) | 1.16 (0.92-1.46) | 0.21 | 1.10 (0.82-1.49) | 1.34 (0.38-4.76) | 1.11 (0.84-1.47) | 0.45 |
|  |  | rs6456723 | 6,286 | 0.93 (0.75-1.15) | 0.81 (0.53-1.24) | 0.92 (0.77-1.08) | 0.31 | 0.85 (0.66-1.10) | 0.87 (0.53-1.42) | 0.89 (0.73-1.09) | 0.26 |
|  |  | rs2273193 | 3,020 | 0.96 (0.78-1.18) | 0.91 (0.61-1.33) | 0.96 (0.82-1.12) | 0.58 | 0.92 (0.72-1.17) | 1.07 (0.69-1.66) | 0.98 (0.82-1.18) | 0.86 |
| 8 | *OSGIN2* | rs2250670 | na | 1.10 (0.89-1.36) | 0.96 (0.69-1.34) | 1.02 (0.88-1.18) | 0.83 | 1.09 (0.84-1.40) | 0.96 (0.65-1.43) | 1.01 (0.85-1.21) | 0.88 |
|  |  | rs2223009 | 1,563 | 1.05 (0.85-1.31) | 0.91 (0.62-1.33) | 0.99 (0.85-1.17) | 0.93 | 1.05 (0.82-1.36) | 0.83 (0.53-1.31) | 0.97 (0.80-1.17) | 0.74 |
|  |  | rs2697671 | 1,871 | 1.06 (0.86-1.30) | 0.96 (0.64-1.43) | 1.02 (0.87-1.19) | 0.85 | 0.96 (0.75-1.23) | 1.07 (0.67-1.71) | 1.00 (0.83-1.20) | 0.99 |
|  |  | rs2697672 | 3,928 | 1.08 (0.77-1.50) | 1.30 (0.36-4.71) | 1.09 (0.81-1.47) | 0.57 | 0.97 (0.65-1.45) | 1.36 (0.30-6.21) | 1.01 (0.70-1.44) | 0.97 |
|  |  | rs2697676 | 11,921 | 0.93 (0.76-1.15) | 0.99 (0.71-1.38) | 0.97 (0.84-1.13) | 0.71 | 0.87 (0.68-1.11) | 1.02 (0.69-1.50) | 0.96 (0.80-1.14) | 0.63 |
|  |  | rs2697677 | 2,816 | 1.02 (0.82-1.27) | 0.71 (0.40-1.25) | 0.95 (0.79-1.14) | 0.58 | 0.93 (0.71-1.21) | 0.69 (0.35-1.37) | 0.89 (0.72-1.11) | 0.30 |
| 10 | *HTRA1* | rs3793917 | na | 0.87 (0.71-1.07) | 0.73 (0.46-1.18) | 0.86 (0.73-1.02) | 0.09 | 0.94 (0.74-1.20) | 0.63 (0.35-1.13) | 0.88 (0.72-1.07) | 0.20 |
|  |  | rs2248799 | 4,669 | 0.89 (0.70-1.13) | 0.98 (0.75-1.30) | 0.99 (0.86-1.14) | 0.89 | 0.96 (0.73-1.27) | 1.04 (0.74-1.44) | 1.02 (0.86-1.20) | 0.84 |
|  |  | rs12571363 | 6,668 | 1.09 (0.84-1.42) | 1.21 (0.48-3.05) | 1.09 (0.87-1.38) | 0.45 | 1.16 (0.86-1.57) | 1.02 (0.31-3.38) | 1.13 (0.86-1.49) | 0.38 |
|  |  | rs932275 | 852 | 0.90 (0.73-1.11) | 0.70 (0.44-1.12) | 0.87 (0.74-1.03) | 0.10 | 0.94 (0.74-1.20) | 0.59 (0.33-1.05) | 0.86 (0.71-1.05) | 0.15 |
|  |  | rs2736914 | 2,038 | 1.11 (0.90-1.38) | 0.95 (0.50-1.79) | 1.07 (0.89-1.29) | 0.48 | 1.07 (0.82-1.38) | 1.04 (0.50-2.17) | 1.05 (0.84-1.31) | 0.65 |
|  |  | rs4752699 | 818 | 0.87 (0.69-1.09) | 1.26 (0.65-2.44) | 0.94 (0.77-1.15) | 0.54 | 1.03 (0.79-1.34) | 1.41 (0.66-2.99) | 1.07 (0.85-1.35) | 0.55 |
|  |  | rs2672590 | 284 | 1.19 (0.97-1.46) | 1.22 (0.80-1.85) | 1.15 (0.98-1.35) | 0.09 | 1.27 (0.99-1.62) | 1.18 (0.72-1.94) | 1.17 (0.97-1.42) | 0.10 |
|  |  | rs7093894 | 276 | 0.94 (0.75-1.17) | 1.15 (0.67-1.96) | 0.99 (0.82-1.19) | 0.89 | 1.03 (0.79-1.35) | 1.44 (0.79-2.64) | 1.10 (0.89-1.36) | 0.38 |
|  |  | rs2672589 | 108 | 1.03 (0.83-1.27) | 1.07 (0.78-1.46) | 1.03 (0.89-1.19) | 0.68 | 0.91 (0.71-1.17) | 0.94 (0.65-1.36) | 0.95 (0.80-1.13) | 0.58 |
|  |  | rs17696741 | 71 | 0.87 (0.68-1.12) | 1.26 (0.59-2.69) | 0.93 (0.75-1.16) | 0.54 | 1.06 (0.80-1.41) | 1.46 (0.61-3.49) | 1.10 (0.86-1.41) | 0.46 |
|  |  | rs2672588 | 237 | 1.12 (0.91-1.37) | 1.15 (0.78-1.68) | 1.09 (0.93-1.28) | 0.27 | 1.10 (0.86-1.40) | 1.07 (0.68-1.70) | 1.06 (0.88-1.28) | 0.51 |
|  |  | rs4752700 | 2,316 | 0.94 (0.75-1.18) | 1.08 (0.81-1.44) | 1.03 (0.89-1.19) | 0.69 | 0.97 (0.74-1.27) | 1.05 (0.74-1.47) | 1.02 (0.86-1.21) | 0.84 |
|  |  | rs760336 | 1,098 | 0.91 (0.72-1.15) | 1.12 (0.84-1.48) | 1.05 (0.91-1.21) | 0.49 | 0.92 (0.69-1.21) | 1.07 (0.77-1.49) | 1.03 (0.87-1.22) | 0.74 |
|  |  | rs2736917 | 273 | 0.86 (0.70-1.07) | 1.14 (0.70-1.83) | 0.95 (0.80-1.12) | 0.53 | 0.83 (0.64-1.07) | 1.15 (0.66-2.00) | 0.93 (0.76-1.14) | 0.48 |
|  |  | rs2284668 | 2,142 | 1.08 (0.87-1.34) | 0.99 (0.60-1.62) | 1.04 (0.88-1.24) | 0.64 | 1.10 (0.85-1.41) | 1.02 (0.57-1.82) | 1.06 (0.86-1.30) | 0.59 |
|  |  | rs2253755 | 705 | 0.99 (0.80-1.22) | 0.94 (0.67-1.32) | 0.98 (0.84-1.14) | 0.76 | 1.06 (0.83-1.36) | 1.04 (0.69-1.55) | 1.03 (0.86-1.23) | 0.73 |
|  |  | rs2300431 | 1,260 | 1.04 (0.84-1.28) | 1.15 (0.77-1.73) | 1.06 (0.90-1.24) | 0.50 | 0.98 (0.77-1.26) | 1.20 (0.76-1.92) | 1.04 (0.86-1.26) | 0.67 |
|  |  | rs2268347 | 4,045 | 0.98 (0.71-1.35) | 1.08 (0.36-3.27) | 1.00 (0.75-1.32) | 0.98 | 0.95 (0.65-1.41) | 0.55 (0.11-2.72) | 0.91 (0.64-1.29) | 0.58 |
|  |  | rs2268348 | 61 | 1.09 (0.83-1.43) | 0.70 (0.23-2.16) | 1.03 (0.81-1.33) | 0.79 | 1.07 (0.77-1.48) | 0.49 (0.10-2.32) | 1.00 (0.74-1.34) | 0.98 |
|  |  | rs2239586 | 2,312 | 1.04 (0.81-1.32) | 0.71 (0.30-1.66) | 0.99 (0.79-1.22) | 0.89 | 0.97 (0.73-1.31) | 0.78 (0.29-2.07) | 0.95 (0.73-1.23) | 0.69 |
|  |  | rs10887154 | 6,105 | 1.09 (0.84-1.42) | 0.77 (0.25-2.32) | 1.05 (0.82-1.33) | 0.71 | 1.05 (0.77-1.43) | 0.51 (0.11-2.44) | 0.99 (0.74-1.32) | 0.93 |
|  |  | rs736960 | 2,796 | 1.09 (0.85-1.41) | 0.63 (0.26-1.51) | 1.01 (0.81-1.26) | 0.92 | 1.11 (0.83-1.49) | 0.70 (0.25-1.96) | 1.04 (0.80-1.34) | 0.78 |
|  |  | rs2672606 | 194 | 1.05 (0.82-1.33) | 0.64 (0.29-1.41) | 0.97 (0.79-1.20) | 0.80 | 0.99 (0.74-1.32) | 0.75 (0.30-1.85) | 0.95 (0.74-1.22) | 0.69 |
|  |  | rs763720 | 4,114 | 1.05 (0.85-1.30) | 0.66 (0.40-1.07) | 0.94 (0.79-1.11) | 0.46 | 1.07 (0.83-1.38) | 0.71 (0.40-1.26) | 0.96 (0.79-1.18) | 0.71 |
|  |  | rs2250804 | 2,434 | 1.08 (0.88-1.33) | 0.72 (0.50-1.03) | 0.93 (0.80-1.08) | 0.34 | 1.14 (0.89-1.45) | 0.71 (0.46-1.10) | 0.95 (0.79-1.13) | 0.55 |
|  |  | rs2268356 | 448 | 1.11 (0.88-1.39) | 0.94 (0.71-1.25) | 0.98 (0.85-1.13) | 0.78 | 1.03 (0.79-1.35) | 0.93 (0.67-1.30) | 0.97 (0.83-1.15) | 0.74 |
|  |  | rs2736928 | 11,691 | 1.08 (0.88-1.33) | 1.07 (0.68-1.70) | 1.06 (0.90-1.25) | 0.49 | 1.01 (0.79-1.30) | 1.01 (0.59-1.74) | 1.01 (0.83-1.23) | 0.92 |
| 15 | *C15orf15* | rs10518801 | na | 0.89 (0.71-1.12) | 0.91 (0.52-1.62) | 0.91 (0.75-1.11) | 0.36 | 0.89 (0.68-1.17) | 0.73 (0.35-1.50) | 0.88 (0.69-1.11) | 0.27 |
|  |  | rs4545742 | 2,125 | 0.99 (0.78-1.27) | 0.48 (0.22-1.06) | 0.90 (0.73-1.11) | 0.32 | 0.92 (0.69-1.23) | 0.63 (0.27-1.51) | 0.88 (0.69-1.13) | 0.30 |
|  |  | rs16976099 | 2,249 | 0.84 (0.59-1.20) | 0.26 (0.03-2.36) | 0.79 (0.57-1.11) | 0.17 | 0.82 (0.53-1.25) | - | 0.74 (0.50-1.11) | 0.15 |
|  |  | rs8038091 | 3,381 | 0.87 (0.64-1.19) | 0.39 (0.10-1.48) | 0.82 (0.62-1.10) | 0.18 | 0.91 (0.63-1.31) | 0.49 (0.10-2.35) | 0.87 (0.62-1.22) | 0.41 |
|  |  | rs8034402 | 4,561 | 0.95 (0.76-1.20) | 0.72 (0.38-1.35) | 0.92 (0.75-1.11) | 0.38 | 0.86 (0.65-1.14) | 0.83 (0.40-1.72) | 0.88 (0.69-1.11) | 0.28 |
|  |  | rs17238164 | 2,957 | 0.87 (0.69-1.10) | 0.89 (0.47-1.69) | 0.89 (0.73-1.09) | 0.26 | 0.84 (0.63-1.11) | 0.97 (0.46-2.05) | 0.89 (0.70-1.12) | 0.31 |
|  |  | rs13733 | 5,214 | 0.82 (0.66-1.02) | 0.86 (0.55-1.35) | 0.87 (0.73-1.04) | 0.12 | 0.79 (0.61-1.02) | 0.86 (0.51-1.47) | 0.85 (0.69-1.05) | 0.14 |
|  |  | rs11855490 | 206 | 0.92 (0.65-1.29) | 0.42 (0.04-4.15) | 0.89 (0.64-1.23) | 0.48 | 0.93 (0.63-1.39) | 0.74 (0.07-7.55) | 0.93 (0.63-1.35) | 0.69 |
|  |  | rs3809540 | 102 | 0.81 (0.65-1.01) | 0.82 (0.53-1.29) | 0.86 (0.72-1.02) | 0.09 | 0.79 (0.60-1.02) | 0.84 (0.49-1.42) | 0.85 (0.69-1.04) | 0.12 |
|  |  | rs3809539 | 202 | 0.94 (0.74-1.21) | 0.55 (0.26-1.19) | 0.88 (0.71-1.09) | 0.24 | 0.87 (0.65-1.17) | 0.72 (0.31-1.66) | 0.86 (0.67-1.11) | 0.25 |
|  |  | rs13380400 | 904 | 0.81 (0.65-1.01) | 0.92 (0.58-1.47) | 0.88 (0.73-1.05) | 0.15 | 0.79 (0.60-1.03) | 0.97 (0.56-1.67) | 0.87 (0.71-1.08) | 0.22 |
|  |  | rs10518804 | 208 | 0.93 (0.73-1.19) | 0.57 (0.24-1.34) | 0.88 (0.71-1.10) | 0.26 | 0.85 (0.63-1.14) | 0.87 (0.35-2.15) | 0.87 (0.67-1.13) | 0.30 |
|  |  | rs2899580 | 845 | 0.82 (0.66-1.02) | 0.92 (0.58-1.47) | 0.88 (0.74-1.06) | 0.17 | 0.79 (0.61-1.03) | 0.96 (0.56-1.66) | 0.88 (0.71-1.08) | 0.22 |
|  |  | rs2899581 | 72 | 0.82 (0.66-1.02) | 0.92 (0.58-1.47) | 0.88 (0.74-1.05) | 0.17 | 0.79 (0.61-1.03) | 0.96 (0.56-1.65) | 0.87 (0.71-1.08) | 0.22 |
|  |  | rs2899582 | 91 | 0.82 (0.66-1.02) | 0.92 (0.58-1.47) | 0.88 (0.74-1.05) | 0.17 | 0.79 (0.61-1.03) | 0.96 (0.56-1.65) | 0.87 (0.71-1.08) | 0.22 |
| 16 | *ZNF200* | rs12917706 | na | 0.92 (0.74-1.15) | 0.85 (0.64-1.15) | 0.92 (0.80-1.07) | 0.28 | 0.88 (0.68-1.14) | 0.88 (0.62-1.24) | 0.93 (0.79-1.10) | 0.39 |
|  |  | rs401298 | 3,096 | 1.00 (0.80-1.24) | 1.10 (0.81-1.48) | 1.03 (0.90-1.20) | 0.64 | 1.01 (0.78-1.31) | 1.12 (0.78-1.60) | 1.05 (0.88-1.24) | 0.59 |
|  |  | rs9927763 | 1,301 | 1.22 (0.91-1.64) | 0.26 (0.06-1.24) | 1.06 (0.81-1.39) | 0.66 | 1.35 (0.96-1.90) | 0.23 (0.03-1.87) | 1.16 (0.85-1.59) | 0.35 |
|  |  | rs2075852 | 330 | 0.91 (0.73-1.15) | 0.96 (0.72-1.27) | 0.97 (0.85-1.12) | 0.71 | 0.84 (0.64-1.10) | 0.92 (0.66-1.28) | 0.95 (0.80-1.12) | 0.53 |
|  |  | rs186493 | 6,726 | 0.77 (0.62-0.95) | 0.76 (0.54-1.06) | **0.83 (0.71-0.97)** | **0.02** | 0.73 (0.57-0.94) | 0.75 (0.50-1.13) | **0.82 (0.68-0.98)** | **0.03** |
| 17 | *MSL1* | rs17678694 | na | 1.02 (0.68-1.54) | 0.79 (0.07-8.95) | 1.01 (0.68-1.48) | 0.97 | 0.75 (0.45-1.26) | - | 0.72 (0.43-1.19) | 0.20 |
|  |  | rs7211770 | 1,933 | 0.85 (0.69-1.04) | 0.74 (0.49-1.10) | **0.85 (0.72-1.00)** | **0.05** | 0.77 (0.60-0.99) | **0.70 (0.44-1.13)** | **0.81 (0.66-0.98)** | **0.03** |
|  | *HEXIM1* | rs1053578 | > 5.0 Mb | 1.23 (0.91-1.67) | 2.25 (0.64-7.86) | 1.28 (0.97-1.69) | 0.08 | 1.33 (0.93-1.89) | 2.86 (0.74-11.0) | **1.40 (1.02-1.91)** | **0.04** |
|  |  | rs8070447 | 8,014 | 1.11 (0.87-1.41) | 0.84 (0.41-1.73) | 1.05 (0.85-1.29) | 0.67 | 1.08 (0.81-1.44) | 0.80 (0.33-1.91) | 1.02 (0.80-1.30) | 0.89 |
|  |  | rs7217422 | 1,166 | 1.08 (0.86-1.35) | 0.84 (0.42-1.67) | 1.03 (0.84-1.25) | 0.79 | 0.98 (0.75-1.28) | 0.94 (0.42-2.09) | 0.98 (0.77-1.23) | 0.83 |
| 19 | *PTPRS* | rs8105746 | na | 0.92 (0.72-1.17) | 1.14 (0.64-2.03) | 0.97 (0.80-1.19) | 0.80 | 1.01 (0.76-1.34) | 1.25 (0.62-2.53) | 1.05 (0.82-1.33) | 0.72 |
|  |  | rs1143700 | 5,919 | 1.07 (0.86-1.34) | 1.01 (0.58-1.77) | 1.05 (0.87-1.26) | 0.61 | 1.02 (0.78-1.32) | 0.97 (0.50-1.88) | 1.00 (0.81-1.25) | 0.98 |
|  |  | rs1978237 | 2,866 | 0.96 (0.79-1.18) | 1.07 (0.72-1.60) | 1.00 (0.85-1.17) | 0.99 | 0.95 (0.75-1.21) | 0.96 (0.59-1.55) | 0.97 (0.80-1.17) | 0.72 |
|  |  | rs2302224 | 981 | 1.08 (0.79-1.47) | 1.40 (0.71-2.75) | 1.12 (0.88-1.44) | 0.35 | 1.03 (0.72-1.48) | 1.06 (0.45-2.48) | 1.03 (0.76-1.38) | 0.86 |
|  |  | rs2230611 | 1,860 | 1.09 (0.85-1.40) | 0.72 (0.26-2.00) | 1.04 (0.83-1.30) | 0.73 | 1.12 (0.83-1.50) | 0.69 (0.19-2.55) | 1.06 (0.81-1.39) | 0.67 |
|  |  | rs11085118 | 1,676 | 0.99 (0.71-1.39) | 1.07 (0.69-1.66) | 1.03 (0.83-1.27) | 0.81 | 1.10 (0.74-1.64) | 0.98 (0.57-1.68) | 1.01 (0.78-1.31) | 0.95 |
|  |  | rs10413063 | 2,125 | 0.95 (0.76-1.18) | 1.10 (0.83-1.47) | 1.03 (0.90-1.19) | 0.66 | 0.93 (0.72-1.21) | 1.16 (0.82-1.62) | 1.05 (0.89-1.24) | 0.55 |
|  |  | rs12975955 | 3,520 | 1.01 (0.81-1.26) | 0.87 (0.65-1.18) | 0.95 (0.82-1.09) | 0.47 | 1.01 (0.78-1.31) | 0.94 (0.66-1.33) | 0.98 (0.82-1.16) | 0.79 |
|  |  | rs10412973 | 1,097 | 1.07 (0.86-1.33) | 0.82 (0.48-1.40) | 1.00 (0.84-1.20) | 1.00 | 1.03 (0.80-1.34) | 0.81 (0.42-1.55) | 0.98 (0.79-1.21) | 0.84 |
|  |  | rs12610082 | 6,108 | 1.12 (0.90-1.38) | 1.46 (0.84-2.53) | 1.15 (0.96-1.38) | 0.13 | 1.05 (0.82-1.36) | 1.76 (0.95-3.25) | 1.15 (0.93-1.42) | 0.19 |
|  |  | rs4807015 | 4,127 | 1.05 (0.83-1.33) | 0.86 (0.65-1.14) | 0.93 (0.81-1.07) | 0.32 | 1.06 (0.80-1.40) | 0.88 (0.63-1.23) | 0.94 (0.80-1.11) | 0.47 |
|  |  | rs2379609 | 5,031 | 1.14 (0.92-1.41) | 1.32 (0.85-2.06) | 1.14 (0.97-1.35) | 0.11 | 1.17 (0.91-1.50) | 1.29 (0.77-2.17) | 1.15 (0.95-1.41) | 0.15 |
|  |  | rs3746130 | 4,226 | 1.12 (0.87-1.45) | 0.60 (0.23-1.54) | 1.03 (0.82-1.29) | 0.81 | 1.14 (0.84-1.53) | 0.30 (0.07-1.34) | 1.00 (0.76-1.31) | 0.98 |
|  |  | rs10415488 | 6,325 | 1.07 (0.86-1.32) | 1.02 (0.76-1.38) | 1.02 (0.89-1.18) | 0.75 | 1.04 (0.81-1.34) | 1.07 (0.76-1.52) | 1.04 (0.88-1.22) | 0.68 |
|  |  | rs886936 | 6,715 | 0.89 (0.70-1.13) | 0.82 (0.62-1.09) | 0.91 (0.79-1.04) | 0.18 | 0.79 (0.60-1.04) | 0.72 (0.52-1.00) | **0.85 (0.72-1.00)** | **0.05** |
|  |  | rs11878779 | 1,286 | 0.84 (0.68-1.04) | 0.78 (0.56-1.09) | 0.87 (0.75-1.01) | 0.06 | 0.75 (0.58-0.97) | 0.67 (0.44-1.00) | **0.79 (0.66-0.95)** | **0.01** |
|  |  | rs17130 | 252 | 1.04 (0.85-1.28) | 1.21 (0.87-1.68) | 1.08 (0.93-1.25) | 0.31 | 1.00 (0.78-1.28) | 1.33 (0.91-1.94) | 1.10 (0.93-1.31) | 0.27 |
|  |  | rs4807016 | 3,520 | 1.09 (0.89-1.34) | 0.92 (0.67-1.27) | 1.00 (0.86-1.15) | 0.99 | 1.09 (0.85-1.39) | 0.86 (0.58-1.27) | 0.98 (0.82-1.16) | 0.78 |
|  |  | rs2238640 | 3,016 | 1.03 (0.84-1.28) | 1.19 (0.87-1.64) | 1.07 (0.93-1.25) | 0.34 | 1.13 (0.88-1.45) | 1.14 (0.78-1.68) | 1.09 (0.91-1.30) | 0.36 |
|  |  | rs1034863 | 6,245 | 1.01 (0.80-1.29) | 0.56 (0.28-1.14) | 0.93 (0.76-1.14) | 0.47 | 0.98 (0.74-1.30) | 0.87 (0.42-1.83) | 0.96 (0.76-1.22) | 0.75 |
|  |  | rs933394 | 1,280 | 0.86 (0.63-1.18) | 0.57 (0.10-3.32) | 0.85 (0.63-1.14) | 0.27 | 0.82 (0.56-1.19) | 0.49 (0.05-4.63) | 0.80 (0.56-1.15) | 0.23 |
|  |  | rs8110570 | 427 | 0.98 (0.77-1.24) | 1.12 (0.57-2.20) | 1.00 (0.82-1.23) | 0.98 | 1.02 (0.77-1.35) | 1.29 (0.61-2.72) | 1.06 (0.83-1.34) | 0.64 |
|  |  | rs1141371 | 4,113 | 0.95 (0.77-1.16) | 0.99 (0.64-1.52) | 0.97 (0.82-1.14) | 0.70 | 0.91 (0.71-1.16) | 0.99 (0.60-1.65) | 0.95 (0.78-1.15) | 0.61 |
|  |  | rs1034917 | 813 | 0.97 (0.77-1.22) | 0.33 (0.13-0.84) | 0.86 (0.70-1.06) | 0.16 | 1.06 (0.81-1.39) | 0.39 (0.13-1.15) | 0.94 (0.74-1.19) | 0.61 |
|  |  | rs7254570 | 4,494 | 1.16 (0.86-1.56) | 2.93 (0.71-12.0) | 1.23 (0.93-1.62) | 0.14 | 1.16 (0.81-1.65) | 2.93 (0.64-13.4) | 1.24 (0.90-1.71) | 0.18 |
|  |  | rs740058 | 9,304 | 0.86 (0.70-1.06) | 0.93 (0.58-1.49) | 0.90 (0.76-1.07) | 0.23 | 0.92 (0.72-1.18) | 0.94 (0.54-1.63) | 0.94 (0.77-1.15) | 0.55 |
|  |  | rs4807711 | 2,173 | 1.02 (0.81-1.28) | 0.49 (0.23-1.03) | 0.92 (0.75-1.12) | 0.42 | 1.12 (0.86-1.46) | 0.51 (0.20-1.29) | 1.00 (0.79-1.26) | 0.98 |
|  |  | rs758512 | 529 | 0.83 (0.59-1.17) | 0.58 (0.21-1.61) | 0.81 (0.60-1.09) | 0.16 | 0.88 (0.59-1.30) | 0.79 (0.27-2.37) | 0.88 (0.63-1.23) | 0.46 |
|  | *CC2D1A* | rs8111004 | > 8.6 Mb | 1.10 (0.89-1.37) | 1.07 (0.70-1.62) | 1.07 (0.90-1.26) | 0.45 | 1.19 (0.92-1.53) | 0.98 (0.59-1.63) | 1.08 (0.89-1.32) | 0.43 |
|  |  | rs3745457 | 4,609 | 1.08 (0.87-1.33) | 1.20 (0.80-1.82) | 1.09 (0.92-1.28) | 0.31 | 1.14 (0.88-1.46) | 1.14 (0.69-1.87) | 1.10 (0.90-1.34) | 0.34 |
|  |  | rs6511901 | 10,878 | 1.12 (0.90-1.39) | 1.13 (0.73-1.74) | 1.09 (0.92-1.30) | 0.32 | 1.21 (0.94-1.56) | 1.11 (0.66-1.86) | 1.13 (0.92-1.38) | 0.24 |
|  |  | rs2305777 | 11,139 | 0.78 (0.63-0.97) | 0.79 (0.54-1.15) | **0.84 (0.72-0.99)** | **0.03** | 0.69 (0.54-0.89) | 0.67 (0.42-1.08) | **0.76 (0.62-0.92)** | **0.005** |
|  |  | rs1059721 | 2,810 | 1.17 (0.94-1.46) | 1.16 (0.61-2.23) | 1.14 (0.95-1.38) | 0.17 | 1.08 (0.83-1.41) | 1.28 (0.62-2.67) | 1.10 (0.88-1.37) | 0.40 |
|  |  | rs2305778 | 2,686 | 0.92 (0.67-1.26) | 0.57 (0.13-2.58) | 0.89 (0.67-1.20) | 0.45 | 0.97 (0.67-1.40) | 1.01 (0.21-4.73) | 0.97 (0.70-1.36) | 0.87 |
|  |  | rs2305779 | 746 | 0.96 (0.75-1.22) | 1.07 (0.40-2.86) | 0.97 (0.78-1.21) | 0.78 | 0.93 (0.70-1.24) | 0.84 (0.24-2.90) | 0.93 (0.71-1.21) | 0.59 |
|  | *PRPF31* | rs4806711 | > 45 Mb | 1.08 (0.87-1.34) | 0.77 (0.44-1.35) | 1.00 (0.84-1.20) | 0.97 | 1.10 (0.86-1.42) | 0.63 (0.31-1.28) | 0.99 (0.80-1.22) | 0.89 |
|  |  | rs12985735 | 4,358 | 1.05 (0.83-1.34) | 1.28 (0.96-1.70) | 1.13 (0.98-1.30) | 0.10 | 1.01 (0.76-1.35) | 1.25 (0.89-1.75) | 1.12 (0.94-1.33) | 0.19 |
|  |  | rs11670086 | 4,076 | 1.07 (0.84-1.36) | 0.84 (0.31-2.26) | 1.04 (0.84-1.29) | 0.72 | 1.15 (0.87-1.51) | 1.27 (0.44-3.62) | 1.14 (0.89-1.47) | 0.30 |
|  |  | rs254272 | 2,072 | 1.04 (0.84-1.29) | 1.13 (0.71-1.81) | 1.05 (0.89-1.25) | 0.56 | 0.99 (0.77-1.29) | 1.37 (0.81-2.33) | 1.08 (0.88-1.32) | 0.47 |
|  |  | rs10424816 | 511 | 0.94 (0.76-1.16) | 1.13 (0.83-1.54) | 1.03 (0.89-1.19) | 0.71 | 0.93 (0.73-1.20) | 1.21 (0.85-1.74) | 1.05 (0.89-1.25) | 0.55 |
|  |  | rs254271 | 549 | 1.12 (0.90-1.38) | 1.09 (0.79-1.49) | 1.06 (0.92-1.23) | 0.41 | 1.14 (0.89-1.47) | 1.03 (0.71-1.51) | 1.05 (0.89-1.25) | 0.56 |
|  |  | rs8102427 | 391 | 0.89 (0.72-1.11) | 1.19 (0.88-1.62) | 1.04 (0.90-1.20) | 0.62 | 0.92 (0.71-1.19) | 1.16 (0.80-1.67) | 1.03 (0.87-1.23) | 0.70 |
|  |  | rs4806716 | 8,720 | 0.96 (0.78-1.19) | 1.48 (0.93-2.36) | 1.07 (0.90-1.26) | 0.45 | 1.00 (0.78-1.28) | 1.84 (1.11-3.06) | 1.16 (0.95-1.41) | 0.14 |

bp to previous represents distance in base pairs between SNPs; AA, common homozygotes; AB, heterozygotes; BB, rare homozygotes; odds ratios, 95% confidence intervals and p-values from logistic regression analysis, adjusted for possible population structure (via principal components), study site, age, body mass index (BMI), hormone therapy, oral contraceptive use, number of live births, age at first live birth, and geographic region; per-allele odds ratios and tests for trend p-values based on an ordinal (log-additive) genotypic response; bold indicates p-value < 0.05.
