# Supplementary material for: Risk of Ovarian Cancer and Inherited Variants in Relapse-Associated Genes
Source: PLoS One. 2010 Jan 27;5(1):e8884. doi: 10.1371/journal.pone.0008884 (PMC2811736; doi:10.1371/journal.pone.0008884)
Supplement: Table S2 — Haplotype results for PRPF31 (global p-value = 0.03) (0.12 MB DOC) [file pone.0008884.s004.doc]

**Table S2. Haplotype results for *PRPF31* (global p-value=0.03)**

|  | **Estimated Haplotype Frequency** | | | **Invasive Disease** | |
| --- | --- | --- | --- | --- | --- |
| **Haplotype** | **Total** | **Patients** | **Controls** | **OR (95% CI)** | **p-value** |
| 11111111 | 0.049 | 0.054 | 0.045 | 1.00 (ref) | NA |
| 10111111 | 0.041 | 0.039 | 0.042 | 0.92 (0.44 - 1.92) | 0.82 |
| 11011111 | 0.044 | 0.046 | 0.042 | 1.43 (0.73 - 2.81) | 0.30 |
| 11111110 | 0.037 | 0.037 | 0.037 | 0.80 (0.41 - 1.54) | 0.50 |
| 01111111 | 0.037 | 0.038 | 0.036 | 1.04 (0.48 - 2.22) | 0.93 |
| 01011111 | 0.033 | 0.034 | 0.032 | 0.81 (0.38 - 1.71) | 0.58 |
| 00011111 | 0.029 | 0.030 | 0.028 | 1.42 (0.66 - 3.09) | 0.37 |
| 00101111 | 0.028 | 0.028 | 0.028 | 0.94 (0.40 - 2.20) | 0.88 |
| 10011111 | 0.026 | 0.024 | 0.027 | 0.68 (0.28 - 1.65) | 0.39 |
| 10011101 | 0.025 | 0.024 | 0.026 | 0.84 (0.37 - 1.86) | 0.66 |
| 00111111 | 0.026 | 0.026 | 0.026 | 0.79 (0.31 - 2.05) | 0.63 |
| 01111101 | 0.024 | 0.024 | 0.024 | 0.50 (0.20 - 1.22) | 0.13 |
| 11101111 | 0.022 | 0.020 | 0.024 | 0.93 (0.35 - 2.45) | 0.88 |
| 11111101 | 0.024 | 0.027 | 0.022 | 2.00 (0.81 - 4.89) | 0.13 |
| 11011110 | 0.021 | 0.021 | 0.021 | 0.95 (0.38 - 2.36) | 0.91 |
| 01101111 | 0.021 | 0.020 | 0.021 | 0.98 (0.37 - 2.55) | 0.96 |
| 10111110 | 0.020 | 0.021 | 0.020 | 1.15 (0.49 - 2.72) | 0.75 |
| 01011101 | 0.020 | 0.020 | 0.020 | 1.09 (0.43 - 2.71) | 0.86 |
| 10011110 | 0.020 | 0.020 | 0.020 | 1.23 (0.53 - 2.84) | 0.63 |
| 10101111 | 0.015 | 0.012 | 0.018 | **0.22 (0.06 - 0.82)** | **0.02** |
| 10111101 | 0.019 | 0.021 | 0.018 | 1.02 (0.42 - 2.49) | 0.96 |
| 00111110 | 0.018 | 0.020 | 0.017 | 1.95 (0.75 - 5.12) | 0.17 |
| 10001111 | 0.015 | 0.013 | 0.016 | 0.74 (0.23 - 2.40) | 0.62 |
| 00111101 | 0.018 | 0.021 | 0.016 | 2.81 (0.85 - 9.22) | 0.09 |
| 11001101 | 0.014 | 0.012 | 0.015 | 1.06 (0.36 - 3.08) | 0.92 |
| 01111011 | 0.013 | 0.012 | 0.013 | 1.18 (0.35 - 3.98) | 0.79 |
| 01001111 | 0.013 | 0.012 | 0.013 | 0.70 (0.23 - 2.13) | 0.53 |
| 10111011 | 0.013 | 0.013 | 0.013 | 1.52 (0.50 - 4.61) | 0.46 |
| 00001111 | 0.014 | 0.015 | 0.013 | 1.53 (0.52 - 4.53) | 0.44 |
| 11001110 | 0.011 | 0.010 | 0.013 | 0.61 (0.20 - 1.82) | 0.37 |
| 11011101 | 0.012 | 0.011 | 0.012 | 0.40 (0.10 - 1.62) | 0.20 |
| 10101101 | 0.013 | 0.015 | 0.012 | 2.86 (0.94 - 8.71) | 0.06 |
| 11011001 | 0.011 | 0.011 | 0.011 | 0.71 (0.24 - 2.08) | 0.54 |
| 00011101 | 0.011 | 0.010 | 0.011 | 0.64 (0.16 - 2.62) | 0.53 |
| 11111011 | 0.010 | 0.010 | 0.011 | 0.90 (0.26 - 3.08) | 0.87 |
| 00101101 | 0.012 | 0.014 | 0.010 | 2.01 (0.54 - 7.55) | 0.30 |
| 11001111 | 0.010 | 0.009 | 0.010 | 0.98 (0.22 - 4.41) | 0.98 |
| 00001101 | 0.010 | 0.010 | 0.010 | 0.83 (0.26 - 2.62) | 0.75 |

For each haplotype, 1 indicates major allele and 0 indicates minor allele at rs4806711, rs12985735, rs11670086, rs254272, rs10424816, rs254271, rs8102427, and 4806716; analyses adjusted for possible population structure (via principal components), age, body mass index (BMI), hormone replacement therapy, oral contraceptive use, number of live births, age at first live birth, and geographic region.
