# Supplementary material for: Risk of Ovarian Cancer and Inherited Variants in Relapse-Associated Genes
Source: PLoS One. 2010 Jan 27;5(1):e8884. doi: 10.1371/journal.pone.0008884 (PMC2811736; doi:10.1371/journal.pone.0008884)
Supplement: Table S3 — Characteristics of study participants by study site (0.14 MB DOC) [file pone.0008884.s005.doc]

**Table S3. Characteristics of study participants by study site**

|  |  | **Mayo Patients (N=339)** | **Mayo Controls (N=469)** | **P-value** | **Duke Patients (N=410)** | **Duke Controls (N=572)** | **P-value** |
| --- | --- | --- | --- | --- | --- | --- | --- |
| Age, years | Mean (S.D.) | 61 (12.5) | 60 (13.0) | 0.29 | 56.2 (10.8) | 54.5 (12.1) | **0.02** |
|  |  |  |  |  |  |  |  |
| Self-reported race | White | 328 (99%) | 460 (99%) | 0.96 | 345 (84%) | 484 (85%) | 0.74 |
|  | African American | 2 (1%) | 2 (<1%) |  | 52 (13%) | 77 (14%) |  |
|  | Asian | 2 (1%) | 2 (<1%) |  | 4 (1%) | 3 (1%) |  |
|  | Other | 1 (<1%) | 1 (<1%) |  | 8 (2%) | 8 (1%) |  |
|  | Missing | 6 | 4 |  | 1 | 0 |  |
|  |  |  |  |  |  |  |  |
| Body mass index, kg/m^2^ | < 23 | 70 (21%) | 109 (25%) | **0.02** | 103 (26%) | 142 (26%) | 0.81 |
|  | 23-26 | 75 (23%) | 122 (28%) |  | 94 (24%) | 125 (23%) |  |
|  | 26-29 | 80 (25%) | 112 (26%) |  | 86 (22%) | 135 (24%) |  |
|  | >29 | 102 (31%) | 95 (22%) |  | 114 (29%) | 153 (28%) |  |
|  | Missing | 12 | 31 |  | 13 | 17 |  |
|  |  |  |  |  |  |  |  |
| Oral contraceptive use | Never | 159 (50%) | 166 (38%) | **<0.001** | 147 (37%) | 180 (32%) | **0.05** |
|  | 1-48 Months | 82 (26%) | 92 (21%) |  | 126 (31%) | 161 (28%) |  |
|  | 48+ Months | 80 (25%) | 174 (40%) |  | 129 (32%) | 226 (40%) |  |
|  | Missing | 18 | 37 |  | 8 | 5 |  |
| Hormone therapy | Never | 199 (62%) | 249 (59%) | 0.69 | 145 (38%) | 339 (63%) | **<0.001** |
|  | 1-60 Months | 58 (18%) | 79 (19%) |  | 137 (36%) | 106 (20%) |  |
|  | 60+ Months | 65 (20%) | 95 (23%) |  | 104 (27%) | 95 (18%) |  |
|  | Missing | 17 | 46 |  | 24 | 32 |  |
|  |  |  |  |  |  |  |  |
| N children/age at first birth | Nulliparous | 56 (17%) | 66 (15%) | 0.29 | 84 (21%) | 75 (13%) | **0.01** |
|  | 1-2/<=20 yrs | 21 (6%) | 25 (6%) |  | 55 (13%) | 72 (13%) |  |
|  | 1-2/>20 yrs | 94 (29%) | 132 (30%) |  | 137 (34%) | 233 (41%) |  |
|  | 3+/<=20 yrs | 63 (19%) | 64 (15%) |  | 71 (17%) | 91 (16%) |  |
|  | 3+/>20 yrs | 96 (29%) | 153 (35%) |  | 62 (15%) | 101 (18%) |  |
|  | Missing | 9 | 29 |  | 1 | 0 |  |
| 1^st^ or 2^nd^ degree family history of ovarian cancer | Yes | 44 (13%) | 33 (7%) | **0.01** | 41 (10%) | 31 (5%) | **0.01** |
|  | No | 286 (87%) | 411 (93%) |  | 369 (90%) | 541 (95%) |  |
|  | Missing | 9 | 25 |  |  |  |  |
| Histologic sub-type | Serous | 208 (62%) | - | - | 244 (60%) | - | - |
|  | Endometrioid | 63 (19%) | - |  | 63 (15%) | - |  |
|  | Clear cell | 23 (7%) | - |  | 32 (8%) | - |  |
|  | Mucinous | 10 (3%) | - |  | 26 (6%) | - |  |
|  | Other | 34 (10%) | - |  | 43 (11%) | - |  |
|  | Missing | 1 | - |  | 2 | - |  |
| Stage | I | 56 (17%) | - | - | 94 (23%) | - | - |
|  | II | 22 (7%) | - |  | 34 (9%) | - |  |
|  | III | 196 (59%) | - |  | 256 (64%) | - |  |
|  | IV | 58 (18%) | - |  | 18 (5%) | - |  |
|  | Missing | 7 | - |  | 8 | - |  |

Data are counts (percentage) unless otherwise indicated. P-values are from t-test for continuous variables and Chi square test for categorical variables.
