# Supplementary material for: Risk of Ovarian Cancer and Inherited Variants in Relapse-Associated Genes
Source: PLoS One. 2010 Jan 27;5(1):e8884. doi: 10.1371/journal.pone.0008884 (PMC2811736; doi:10.1371/journal.pone.0008884)
Supplement: Table S4 — SNP and genotype information (0.56 MB DOC) [file pone.0008884.s006.doc]

**Table S4. SNP and genotype i**nformation

|  |  |  |  |  | **Location (bp to gene)** | **Selection** | **Call Rate** | **HWE p-value** |  | **N Patients** | | | **N Controls** | | |
| --- | --- | --- | --- | --- | --- | --- | --- | --- | --- | --- | --- | --- | --- | --- | --- |
| **Chr** | **Gene** | **rsid** | **Position** | **Alleles** |  |  |  |  | **MAF** | **AA** | **AB** | **BB** | **AA** | **AB** | **BB** |
| 1 | *SF3A3* | rs17465651 | 38,191,083 | G/A | 3′ (4,151) | tagSNP (bin 4) | 0.998 | 0.48 | 0.42 | 260 | 347 | 140 | 352 | 505 | 182 |
|  |  | rs17465826 | 38,196,108 | A/C | 3′ UTR | tagSNP (bin 6) & pfSNP | 0.998 | 1.00 | 0.04 | 690 | 56 | 1 | 959 | 80 | 1 |
|  |  | rs1050257 | 38,196,175 | A/G | 3′ UTR | tagSNP (bin 1) & pfSNP | 0.993 | 0.97 | 0.13 | 576 | 154 | 9 | 785 | 235 | 19 |
|  |  | rs9293 | 38,196,440 | G/C | 3′ UTR | pfSNP | 0.451 | 0.77 | 0.13 | 259 | 78 | 2 | 356 | 106 | 7 |
|  |  | rs6660034 | 38,216,920 | G/A | intron | tagSNP (bin 1) | 0.999 | 0.78 | 0.13 | 582 | 156 | 10 | 792 | 230 | 19 |
|  |  | rs7512424 | 38,219,853 | A/G | intron | tagSNP (bin 5) | 0.999 | 0.78 | 0.13 | 581 | 157 | 10 | 792 | 230 | 19 |
|  |  | rs7528185 | 38,224,646 | G/A | intron | tagSNP (bin 3) | 0.997 | **0.02** | 0.27 | 391 | 286 | 68 | 543 | 436 | 60 |
|  |  | rs4072980 | 38,228,693 | G/A | 5′ (345) | tagSNP (bin 2) & pfSNP | 0.996 | 0.48 | 0.42 | 253 | 348 | 145 | 342 | 519 | 176 |
| 4 | *MFSD7* | rs7690350 | 670,843 | A/G | Intron | tagSNP (bin 1) | 0.434 | 0.66 | 0.19 | 215 | 100 | 9 | 297 | 140 | 15 |
|  |  | rs6840253 | 676,563 | A/G | 5′ (3,590) | tagSNP (bin 2) & pfSNP | 0.998 | 0.29 | 0.13 | 591 | 146 | 12 | 785 | 228 | 24 |
|  |  | rs4690290 | 679,502 | G/C | 5′ (6,529) | pfSNP | 0.996 | 0.48 | 0.48 | 199 | 369 | 179 | 278 | 526 | 231 |
| 6 | *ID4* | rs9348399 | 19,935,884 | G/A | 5′ (9,712) | pfSNP | 0.998 | 0.48 | 0.15 | 542 | 196 | 10 | 752 | 268 | 19 |
|  |  | rs6926229 | 19,936,192 | G/A | 5′ (9,404) | pfSNP | 0.997 | 0.46 | 0.43 | 228 | 383 | 135 | 326 | 524 | 189 |
|  |  | rs6456278 | 19,939,194 | T/A | 5′ (6,402) | pfSNP | 0.997 | 0.36 | 0.49 | 192 | 350 | 205 | 272 | 507 | 259 |
|  |  | rs6922714 | 19,939,539 | G/A | 5′ (6,057) | pfSNP | 0.998 | 0.48 | 0.35 | 332 | 327 | 88 | 449 | 462 | 128 |
|  |  | rs12528525 | 19,939,797 | A/C | 5′ (5,799) | pfSNP | 0.998 | 0.86 | 0.30 | 387 | 288 | 71 | 507 | 439 | 94 |
|  |  | rs1980461 | 19,942,988 | G/C | 5′ (2,608) | tagSNP (bin 1) & pfSNP | 0.999 | 0.27 | 0.50 | 199 | 352 | 198 | 268 | 510 | 262 |
|  |  | rs8214 | 19,947,758 | G/A | 3′ UTR | pfSNP | 0.999 | 0.41 | 0.22 | 489 | 229 | 30 | 642 | 342 | 57 |
|  |  | rs1047033 | 19,948,471 | A/G | 3′ UTR | tagSNP (bin 2) & pfSNP | 0.998 | 0.54 | 0.29 | 359 | 314 | 75 | 529 | 414 | 96 |
|  |  | rs6906699 | 19,952,334 | A/G | 3′ (3,440) | tagSNP (bin 4) | 0.999 | 0.18 | 0.23 | 443 | 267 | 39 | 630 | 350 | 60 |
|  | *BTN3A3* | rs4711110 | 26,543,792 | G/A | 5′ (4,950) | tagSNP (bin 2) & pfSNP | 0.997 | 0.17 | 0.13 | 573 | 164 | 11 | 783 | 231 | 23 |
|  |  | rs12206812 | 26,544,571 | G/A | 5′ (4,171) | pfSNP | 0.450 | 0.78 | 0.18 | 239 | 96 | 4 | 313 | 140 | 14 |
|  |  | rs10456330 | 26,544,671 | G/A | 5′ (4,071) | tagSNP (bin 5) & pfSNP | 0.999 | 0.66 | 0.08 | 609 | 131 | 9 | 872 | 158 | 9 |
|  |  | rs12208390 | 26,545,085 | G/A | 5′ (3,657) | pfSNP | 1.000 | 0.45 | 0.09 | 609 | 131 | 9 | 873 | 158 | 10 |
|  |  | rs9379874 | 26,548,408 | A/T | 5′ (334) | pfSNP | 0.992 | 0.48 | 0.47 | 229 | 334 | 180 | 295 | 512 | 225 |
|  |  | rs17539219 | 26,548,521 | A/C | 5′ (221) | tagSNP (bin 1) & pfSNP | 0.999 | 0.66 | 0.18 | 505 | 226 | 18 | 707 | 300 | 32 |
|  |  | rs12214444 | 26,548,631 | G/A | 5′ (111) | pfSNP | 0.999 | 0.18 | 0.13 | 573 | 165 | 10 | 785 | 232 | 23 |
|  |  | rs13220495 | 26,549,619 | G/A | intron | tagSNP (bin 9) | 0.999 | 0.07 | 0.09 | 620 | 122 | 5 | 868 | 159 | 14 |
|  |  | rs9379875 | 26,552,711 | G/A | intron | tagSNP (bin 7) | 0.998 | 0.21 | 0.11 | 595 | 141 | 10 | 821 | 201 | 18 |
|  |  | rs3846845 | 26,552,894 | G/A | intron | tagSNP (bin 3) | 0.990 | 0.74 | 0.46 | 231 | 327 | 182 | 302 | 506 | 224 |
|  |  | rs1796524 | 26,557,198 | G/A | intron | tagSNP (bin 8) | 0.989 | 0.50 | 0.10 | 590 | 138 | 10 | 843 | 183 | 7 |
|  |  | rs6456723 | 26,563,484 | C/G | 3′ (1,862) | tagSNP (bin 4) | 0.999 | 0.68 | 0.25 | 439 | 261 | 47 | 596 | 367 | 78 |
|  |  | rs2273193 | 26,566,504 | C/G | 3′ (4,882) | tagSNP (bin 6) | 0.996 | 0.29 | 0.26 | 418 | 274 | 52 | 579 | 382 | 78 |
| 8 | *OSGIN2* | rs2250670 | 90,983,445 | A/G | 5′ UTR | tagSNP (bin 5) & pfSNP | 0.996 | **0.01** | 0.32 | 362 | 292 | 92 | 508 | 389 | 139 |
|  |  | rs2223009 | 90,985,008 | G/C | Intron | tagSNP (bin 6) | 0.996 | **0.01** | 0.28 | 419 | 249 | 79 | 570 | 345 | 121 |
|  |  | rs2697671 | 90,986,879 | A/G | Intron | tagSNP (bin 3) | 0.999 | 0.11 | 0.24 | 439 | 263 | 46 | 609 | 362 | 70 |
|  |  | rs2697672 | 90,990,807 | G/A | Intron | tagSNP (bin 8) & pfSNP | 0.999 | 0.20 | 0.05 | 669 | 74 | 5 | 938 | 97 | 5 |
|  |  | rs2697676 | 91002728 | A/G | Intron | tagSNP (bin 2) | 0.998 | 0.74 | 0.32 | 365 | 303 | 79 | 491 | 440 | 109 |
|  |  | rs2697677 | 91,005,544 | G/A | Intron | tagSNP (bin 7) | 0.999 | 0.15 | 0.18 | 522 | 205 | 20 | 709 | 291 | 41 |
|  |  | rs1881469 | 91,010,075 | A/T | 3′ (804) | tagSNP (bin 1) | Failed (no amplification) | | | | | | | | |
| 10 | *HTRA1* | rs3793917 | 124,209,265 | G/C | 5′ (1,782) | pfSNP | 0.993 | 0.85 | 0.23 | 467 | 247 | 32 | 606 | 372 | 53 |
|  |  | rs2248799 | 124,213,934 | G/A | Intron | tagSNP (bin 8) | 0.996 | 0.56 | 0.50 | 200 | 364 | 184 | 261 | 521 | 253 |
|  |  | rs12571363 | 124220602 | C/A | Intron | tagSNP (bin 24) | 0.998 | 0.59 | 0.09 | 605 | 132 | 9 | 855 | 175 | 11 |
|  |  | rs932275 | 124,221,454 | G/A | Intron | tagSNP (bin 9) | 0.996 | 0.83 | 0.23 | 468 | 245 | 32 | 617 | 364 | 57 |
|  |  | rs2736914 | 124,223,492 | C/G | Intron | tagSNP (bin 18) | 0.998 | 0.70 | 0.16 | 509 | 220 | 18 | 723 | 292 | 25 |
|  |  | rs4752699 | 124,224,310 | G/A | Intron | tagSNP (bin 22) | 0.998 | 0.73 | 0.15 | 556 | 173 | 19 | 754 | 265 | 20 |
|  |  | rs2672590 | 124,224,594 | A/C | Intron | tagSNP (bin 14) | 0.998 | 0.88 | 0.24 | 401 | 298 | 47 | 595 | 385 | 61 |
|  |  | rs7093894 | 124,224,870 | C/A | Intron | tagSNP (bin 21) | 0.995 | 0.90 | 0.17 | 526 | 192 | 28 | 716 | 284 | 35 |
|  |  | rs2672589 | 124,224,978 | A/G | Intron | tagSNP (bin 15) | 0.982 | 0.69 | 0.36 | 289 | 348 | 99 | 416 | 473 | 132 |
|  |  | rs17696741 | 124,225,049 | G/A | Intron | tagSNP (bin 23) | 0.998 | 0.82 | 0.12 | 591 | 143 | 14 | 800 | 223 | 15 |
|  |  | rs2672588 | 124,225,286 | A/G | Intron | tagSNP (bin 13) | 0.998 | 0.85 | 0.27 | 378 | 314 | 57 | 545 | 418 | 75 |
|  |  | rs4752700 | 124,227,602 | A/G | Intron | tagSNP (bin 5) | 0.997 | 0.62 | 0.45 | 224 | 355 | 169 | 312 | 508 | 217 |
|  |  | rs760336 | 124,228,700 | A/G | Intron | tagSNP (bin 4) | 0.994 | 0.47 | 0.48 | 200 | 353 | 192 | 278 | 516 | 241 |
|  |  | rs2736917 | 124,228,973 | A/G | Intron | tagSNP (bin 20) | 0.998 | 0.94 | 0.20 | 494 | 217 | 35 | 669 | 328 | 44 |
|  |  | rs2284668 | 124,230,842 | A/G | Intron | tagSNP (bin 2) | 0.999 | 0.18 | 0.19 | 484 | 233 | 31 | 691 | 306 | 44 |
|  |  | rs2253755 | 124,231,547 | A/G | Intron | tagSNP (bin 12) | 0.997 | 0.59 | 0.31 | 370 | 306 | 71 | 504 | 428 | 106 |
|  |  | rs2300431 | 124,232,807 | G/A | Intron | tagSNP (bin 19) | 0.999 | 0.91 | 0.25 | 414 | 283 | 51 | 589 | 388 | 64 |
|  |  | rs2268347 | 124,236,852 | G/A | Intron | tagSNP (bin 7) | 0.996 | **0.01** | 0.06 | 663 | 75 | 6 | 920 | 110 | 8 |
|  |  | rs2268348 | 124,236,913 | G/A | Intron | tagSNP (bin 26) | 0.998 | 0.23 | 0.08 | 626 | 116 | 5 | 884 | 146 | 10 |
|  |  | rs2239586 | 124,239,225 | G/A | Intron | tagSNP (bin 1) | 0.999 | 0.31 | 0.11 | 587 | 153 | 9 | 819 | 204 | 17 |
|  |  | rs10887154 | 124,245,330 | A/G | Intron | tagSNP (bin 3) | 0.997 | 0.41 | 0.09 | 614 | 129 | 5 | 862 | 164 | 10 |
|  |  | rs736960 | 124,248,126 | A/G | Intron | tagSNP (bin 25) | 0.998 | 0.20 | 0.11 | 589 | 151 | 8 | 833 | 188 | 18 |
|  |  | rs2672606 | 124,248,320 | A/C | Intron | tagSNP (bin 1) | 1.000 | 0.20 | 0.12 | 580 | 159 | 10 | 809 | 210 | 22 |
|  |  | rs763720 | 124,252,434 | G/A | Intron | tagSNP (bin 16) | 0.996 | 0.08 | 0.21 | 472 | 245 | 28 | 658 | 322 | 57 |
|  |  | rs2250804 | 124,254,868 | A/G | Intron | tagSNP (bin 6) | 0.996 | 0.57 | 0.32 | 349 | 337 | 59 | 480 | 442 | 116 |
|  |  | rs2268356 | 124,255,316 | A/G | Intron | tagSNP (bin 11) | 0.998 | 0.67 | 0.46 | 216 | 385 | 146 | 305 | 512 | 222 |
|  |  | rs2736928 | 124,267,007 | A/G | 3′ (2,594) | tagSNP (bin 10) | 0.997 | 0.76 | 0.22 | 438 | 272 | 37 | 632 | 357 | 49 |
| 15 | *C15orf15* | rs10518801 | 53,255,909 | G/A | 3′ (4,895) | tagSNP (bin 5) | 0.999 | 0.15 | 0.17 | 537 | 189 | 23 | 724 | 280 | 36 |
|  |  | rs4545742 | 53,258,034 | A/G | 3′ (2,770) | tagSNP (bin 4) | 1.000 | **0.01** | 0.13 | 580 | 160 | 9 | 797 | 217 | 27 |
|  |  | rs16976099 | 53,260,283 | T/A | 3′ (521) | tagSNP (bin 4) | 1.000 | 0.13 | 0.05 | 690 | 58 | 1 | 944 | 92 | 5 |
|  |  | rs8038091 | 53,263,664 | A/G | Intron | tagSNP (bin 3) | 0.999 | 0.41 | 0.07 | 656 | 88 | 3 | 900 | 131 | 10 |
|  |  | rs8034402 | 53,268,225 | A/G | Intron | tagSNP (bin 2) | 1.000 | **0.005** | 0.15 | 556 | 177 | 16 | 757 | 252 | 32 |
|  |  | rs17238164 | 53,271,182 | A/G | Intron | tagSNP (bin 6) | 0.999 | 0.45 | 0.14 | 569 | 162 | 17 | 770 | 245 | 26 |
|  |  | rs13733 | 53,276,396 | A/G | 5′ UTR | pfSNP | 0.998 | 0.16 | 0.23 | 483 | 225 | 41 | 632 | 342 | 64 |
|  |  | rs11855490 | 53,276,602 | G/A | 5′ (79) | pfSNP | 0.999 | 0.89 | 0.05 | 681 | 67 | 1 | 938 | 98 | 3 |
|  |  | rs3809540 | 53,276,704 | A/C | 5′ (181) | tagSNP (bin 1) & pfSNP | 0.998 | 0.13 | 0.23 | 484 | 223 | 41 | 631 | 341 | 66 |
|  |  | rs3809539 | 53,276,906 | A/C | 5′ (383) | pfSNP | 0.998 | **0.01** | 0.13 | 591 | 148 | 10 | 802 | 210 | 25 |
|  |  | rs13380400 | 53,277,810 | A/G | 5′ (1,287) | pfSNP | 0.997 | 0.48 | 0.22 | 488 | 220 | 40 | 639 | 340 | 58 |
|  |  | rs10518804 | 53,278,018 | G/A | 5′ (1,495) | pfSNP | 0.998 | 0.11 | 0.12 | 593 | 147 | 8 | 808 | 212 | 19 |
|  |  | rs2899580 | 53,278,863 | A/G | 5′ (2,340) | pfSNP | 0.997 | 0.46 | 0.22 | 487 | 222 | 40 | 639 | 339 | 58 |
|  |  | rs2899581 | 53,278,935 | A/G | 5′ (2,412) | pfSNP | 0.998 | 0.48 | 0.22 | 487 | 222 | 40 | 639 | 340 | 58 |
|  |  | rs2899582 | 53,279,026 | A/G | 5′ (2,503) | pfSNP | 0.998 | 0.48 | 0.22 | 487 | 222 | 40 | 639 | 340 | 58 |
| 16 | *ZNF200* | rs12917706 | 3,217,879 | C/G | Intron | tagSNP (bin 1) | 0.998 | 0.39 | 0.43 | 256 | 370 | 122 | 335 | 519 | 185 |
|  |  | rs401298 | 3,220,975 | G/A | Intron | tagSNP (bin 4) | 0.999 | 0.76 | 0.37 | 300 | 336 | 112 | 422 | 475 | 143 |
|  |  | rs9927763 | 3,222,276 | C/A | Intron | tagSNP (bin 3) | 0.998 | 0.08 | 0.07 | 639 | 107 | 2 | 906 | 121 | 11 |
|  |  | rs2075852 | 3,222,606 | A/G | Intron | tagSNP (bin 2) | 0.996 | 0.36 | 0.47 | 216 | 370 | 159 | 289 | 526 | 223 |
|  |  | rs186493 | 3,229,332 | A/C | 5′ (3,922) | tagSNP (bin 5) & pfSNP | 0.999 | 0.97 | 0.32 | 396 | 281 | 71 | 484 | 445 | 111 |
| 17 | *MSL1* | rs17678694 | 35,540,596 | C/G | Intron | tagSNP (bin 2) | 0.998 | 0.32 | 0.03 | 700 | 46 | 1 | 974 | 64 | 2 |
|  |  | rs7211770 | 35,542,529 | A/G | Intron | tagSNP (bin 1) | 0.997 | 0.27 | 0.28 | 418 | 276 | 54 | 539 | 407 | 91 |
|  | *HEXIM1* | rs1053578 | 40,577,068 | A/G | 5′ (3,399) | tagSNP (bin 3) & pfSNP | 0.994 | 0.77 | 0.06 | 643 | 94 | 7 | 922 | 109 | 4 |
|  |  | rs8070447 | 40,585,082 | A/C | 3′ UTR | tagSNP (bin 2) & pfSNP | 0.998 | 0.16 | 0.12 | 576 | 160 | 12 | 809 | 206 | 24 |
|  |  | rs7217422 | 40,586,248 | C/G | 3′ (997) | tagSNP (bin 1) | 0.999 | 0.14 | 0.14 | 545 | 190 | 14 | 773 | 241 | 25 |
| 19 | *PTPRS* | rs8105746 | 5,151,856 | G/A | 3′ (4,663) | tagSNP (bin 17) | 0.999 | 0.59 | 0.15 | 558 | 164 | 27 | 763 | 245 | 31 |
|  |  | rs1143700 | 5,157,775 | G/A | 3′ UTR | tagSNP (bin 42) & pfSNP | 0.998 | 0.67 | 0.17 | 506 | 217 | 24 | 729 | 278 | 33 |
|  |  | rs1978237 | 5,160,641 | C/G | Intron | tagSNP (bin 10) | 0.998 | 0.13 | 0.27 | 389 | 305 | 52 | 546 | 426 | 68 |
|  |  | rs2302224 | 5,161,622 | G/A | Intron | tagSNP (bin 44) | 0.448 | 0.25 | 0.24 | 184 | 133 | 19 | 265 | 179 | 22 |
|  |  | rs2230611 | 5,163,482 | G/A | Synonymous | tagSNP (bin 51) | 0.999 | 0.68 | 0.10 | 593 | 149 | 6 | 837 | 191 | 12 |
|  |  | rs11085118 | 5,165,158 | G/A | Intron | tagSNP (bin 28) | 0.450 | 0.68 | 0.42 | 105 | 171 | 61 | 155 | 231 | 82 |
|  |  | rs10413063 | 5,167,283 | C/G | Intron | tagSNP (bin 31) | 0.996 | 0.43 | 0.42 | 265 | 323 | 158 | 363 | 473 | 200 |
|  |  | rs12975955 | 5,170,803 | A/G | Intron | tagSNP (bin 8) | 0.997 | 0.86 | 0.42 | 255 | 378 | 115 | 349 | 501 | 187 |
|  |  | rs10412973 | 5,171,900 | G/A | Intron | tagSNP (bin 36) | 0.999 | 0.39 | 0.18 | 499 | 226 | 24 | 707 | 293 | 40 |
|  |  | rs12610082 | 5,178,008 | A/C | Intron | tagSNP (bin 43) | 0.998 | 0.25 | 0.18 | 479 | 242 | 28 | 698 | 310 | 29 |
|  |  | rs4807015 | 5,182,135 | A/G | Intron | tagSNP (bin 24) | 0.993 | 0.09 | 0.47 | 228 | 358 | 159 | 310 | 469 | 253 |
|  |  | rs2379609 | 5,187,166 | A/G | Intron | tagSNP (bin 39) | 0.994 | 0.47 | 0.22 | 427 | 272 | 45 | 634 | 354 | 48 |
|  |  | rs3746130 | 5,191,392 | G/A | Intron | tagSNP (bin 52) | 0.994 | **0.04** | 0.10 | 601 | 135 | 7 | 848 | 174 | 15 |
|  |  | rs10415488 | 5,197,717 | A/G | Intron | tagSNP (bin 26) | 0.989 | 0.32 | 0.38 | 274 | 357 | 113 | 403 | 468 | 155 |
|  |  | rs886936 | 5,204,432 | G/A | Intron | tagSNP (bin 21) | 0.995 | 0.99 | 0.50 | 204 | 369 | 171 | 259 | 517 | 261 |
|  |  | rs11878779 | 5,205,718 | G/A | Intron | tagSNP (bin 11) | 0.997 | 0.55 | 0.33 | 381 | 291 | 74 | 480 | 437 | 122 |
|  |  | rs17130 | 5,205,970 | A/G | Intron | tagSNP (bin 34) | 0.996 | 0.94 | 0.32 | 323 | 335 | 89 | 470 | 460 | 106 |
|  |  | rs4807016 | 5,209,490 | G/A | Intron | tagSNP (bin 33) | 0.998 | 0.21 | 0.33 | 330 | 335 | 84 | 470 | 443 | 125 |
|  |  | rs2238640 | 5,212,506 | A/G | Intron | tagSNP (bin 3) | 0.993 | 0.81 | 0.34 | 319 | 317 | 108 | 458 | 440 | 136 |
|  |  | rs1034863 | 5,218,751 | G/A | Intron | tagSNP (bin 54) | 0.996 | 0.97 | 0.14 | 567 | 166 | 13 | 779 | 230 | 28 |
|  |  | rs933394 | 5,220,031 | C/G | Intron | tagSNP (bin 62) | 0.999 | 0.87 | 0.06 | 672 | 75 | 2 | 916 | 119 | 4 |
|  |  | rs8110570 | 5,220,458 | A/G | Intron | tagSNP (bin 50) | 0.996 | 0.91 | 0.14 | 561 | 165 | 18 | 778 | 240 | 21 |
|  |  | rs1141371 | 5,224,571 | A/C | Synonymous | tagSNP (bin 37) | 0.996 | 0.94 | 0.23 | 452 | 255 | 42 | 611 | 367 | 56 |
|  |  | rs1034917 | 5,225,384 | G/A | Intron | tagSNP (bin 58) | 0.996 | 0.25 | 0.14 | 565 | 174 | 6 | 773 | 239 | 25 |
|  |  | rs7254570 | 5,229,878 | A/C | Intron | tagSNP (bin 1) | 0.999 | 0.84 | 0.06 | 646 | 95 | 7 | 922 | 116 | 3 |
|  |  | rs740058 | 5,239,182 | A/C | Intron | tagSNP (bin 4) | 0.998 | **0.04** | 0.24 | 454 | 260 | 34 | 592 | 399 | 48 |
|  |  | rs4807711 | 5,241,355 | C/G | Intron | tagSNP (bin 14) | 0.998 | 0.46 | 0.15 | 550 | 189 | 10 | 761 | 247 | 30 |
|  |  | rs758512 | 5,241,884 | C/G | Intron | tagSNP (bin 48) | 0.451 | 0.99 | 0.17 | 250 | 83 | 6 | 326 | 129 | 13 |
|  | *CC2D1A* | rs8111004 | 13,873,165 | A/G | 5′ (4,887) | tagSNP (bin 1) & pfSNP | 0.996 | 0.71 | 0.23 | 434 | 258 | 55 | 623 | 340 | 72 |
|  |  | rs3745457 | 13,877,774 | G/A | 5′ (278) | tagSNP (bin 5) & pfSNP | 0.996 | 0.93 | 0.23 | 428 | 264 | 55 | 613 | 358 | 64 |
|  |  | rs6511901 | 13,888,652 | G/A | Intron | tagSNP (bin 2) | 0.999 | 0.88 | 0.24 | 431 | 248 | 70 | 621 | 327 | 91 |
|  |  | rs2305777 | 13,899,791 | G/A | Missense T801M | tagSNP (bin 3) & pfSNP | 0.999 | 0.95 | 0.27 | 438 | 256 | 55 | 558 | 399 | 83 |
|  |  | rs2290664 | 13,902,371 | C/G | 3′ UTR | pfSNP | Failed (cluster compression) | | | | | | | | |
|  |  | rs1059721 | 13,902,601 | G/A | 3′ UTR | tagSNP (bin 4) & pfSNP | 0.997 | 0.94 | 0.16 | 513 | 217 | 17 | 738 | 275 | 24 |
|  |  | rs2305778 | 13,905,287 | G/A | 3′ (2,595) | tagSNP (bin 7) | 0.994 | 0.59 | 0.06 | 666 | 79 | 3 | 908 | 119 | 5 |
|  |  | rs2305779 | 13,906,033 | G/A | 3′ (3,341) | tagSNP (bin 6) | 0.998 | 0.15 | 0.12 | 583 | 157 | 8 | 805 | 224 | 10 |
|  | *PRPF31* | rs4806711 | 59,311,003 | A/G | Intron | tagSNP (bin 9) | 0.998 | 0.74 | 0.19 | 488 | 239 | 22 | 691 | 306 | 40 |
|  |  | rs12985735 | 59,315,361 | G/A | Intron | tagSNP (bin 2) | 0.995 | 0.34 | 0.46 | 196 | 359 | 190 | 299 | 511 | 226 |
|  |  | rs11670086 | 59,319,437 | G/A | Intron | tagSNP (bin 1) | 0.998 | 0.49 | 0.11 | 576 | 165 | 7 | 812 | 215 | 11 |
|  |  | rs254272 | 59,321,509 | A/G | intron | tagSNP (bin 10) | 0.995 | 0.19 | 0.20 | 490 | 221 | 35 | 676 | 312 | 47 |
|  |  | rs10424816 | 59,322,020 | A/C | intron | tagSNP (bin 7) | 0.992 | 0.44 | 0.34 | 334 | 306 | 102 | 452 | 454 | 128 |
|  |  | rs254271 | 59,322,569 | G/C | intron | tagSNP (bin 5) | 0.996 | 0.83 | 0.34 | 315 | 336 | 95 | 461 | 449 | 127 |
|  |  | rs8102427 | 59,322,960 | G/A | intron | tagSNP (bin 4) | 0.994 | 0.66 | 0.34 | 326 | 308 | 111 | 451 | 462 | 122 |
|  |  | rs4806716 | 59,331,680 | A/G | 3′ (4,726) | tagSNP (bin 8) | 0.991 | 0.19 | 0.22 | 454 | 245 | 43 | 622 | 368 | 41 |

Position from genome build 36.3; Refseq release 29 (May 4, 2008) Call rate, among all participants; MAF calculated using all controls; HWE p-value calculated using White non-Hispanic controls only, bold indicates p < 0.05; AA, common homozygotes; AB, heterozygotes; BB, rare homozygotes; WGA failed for *BTN3A3* rs12206812, *PTPRS* rs2302224, and *MFSD7* rs7690350 due to poor clustering, *SF3A3* rs9293 due to cluster compression, and *PTPRS* rs758512 and rs11085118 due to no amplification; tagSNP indicates tagging SNPs within LD bin listed (bin 1 is largest); pfSNP indicates chosen for putative function based on location relative to gene (all SNPs with MAF > 0.05 in UTRs, within 1 kb 5′ upstream, and missense SNPs were included).
